# Mechanisms of polymyxin resistance: acquired and intrinsic resistance in bacteria

**DOI:** 10.3389/fmicb.2014.00643

**Published:** 2014-11-26

**Authors:** Abiola O. Olaitan, Serge Morand, Jean-Marc Rolain

**Affiliations:** ^1^Unité de Recherche sur les Maladies Infectieuses et Tropicales Emergentes CNRS-IRD UMR 6236, Méditerranée Infection, Faculté de Médecine et de Pharmacie, Aix-Marseille-UniversitéMarseille, France; ^2^Institut des Sciences de l'Evolution, CNRS-IRD-UM2, CC065, Université Montpellier 2Montpellier, France

**Keywords:** lipopolysaccharides, lipid A, Enterobacteriaceae, non-fermentative bacilli, mutation, two-component systems, antibiotic resistance

## Abstract

Polymyxins are polycationic antimicrobial peptides that are currently the last-resort antibiotics for the treatment of multidrug-resistant, Gram-negative bacterial infections. The reintroduction of polymyxins for antimicrobial therapy has been followed by an increase in reports of resistance among Gram-negative bacteria. Some bacteria, such as *Klebsiella pneumoniae*, *Pseudomonas aeruginosa*, and *Acinetobacter baumannii*, develop resistance to polymyxins in a process referred to as acquired resistance, whereas other bacteria, such as *Proteus* spp., *Serratia* spp., and *Burkholderia* spp., are naturally resistant to these drugs. Reports of polymyxin resistance in clinical isolates have recently increased, including acquired and intrinsically resistant pathogens. This increase is considered a serious issue, prompting concern due to the low number of currently available effective antibiotics. This review summarizes current knowledge concerning the different strategies bacteria employ to resist the activities of polymyxins. Gram-negative bacteria employ several strategies to protect themselves from polymyxin antibiotics (polymyxin B and colistin), including a variety of lipopolysaccharide (LPS) modifications, such as modifications of lipid A with phosphoethanolamine and 4-amino-4-deoxy-L-arabinose, in addition to the use of efflux pumps, the formation of capsules and overexpression of the outer membrane protein OprH, which are all effectively regulated at the molecular level. The increased understanding of these mechanisms is extremely vital and timely to facilitate studies of antimicrobial peptides and find new potential drugs targeting clinically relevant Gram-negative bacteria.

## Introduction

Recently, the rise in infections caused by multidrug-resistant (MDR) Gram-negative bacteria, especially the resistance to carbapenems most importantly observed in *Pseudomonas aeruginosa*, *Acinetobacter baumannii*, *Klebsiella pneumoniae*, and *Escherichia coli*, has led to the resuscitation of polymyxins (polymyxin B and colistin) worldwide as a last-resort treatment option (Stein and Raoult, 2002; Falagas and Michalopoulos, 2006; Biswas et al., 2012). Resistance to polymyxins by bacteria that are normally susceptible to these drugs has been reported (Johansen et al., 2008; Arduino et al., 2012; Mammina et al., 2012). There are also reports of increases in infections caused by naturally polymyxin-resistant bacteria, such as *Proteus*, *Providencia, Morganella*, and *Serratia* (Hayakawa et al., 2012; Merkier et al., 2013; Samonis et al., 2014).

Bacteria employ several means to protect themselves from adverse environmental stimuli, including exposure to cationic antimicrobial peptides, such as polymyxin B and colistin. These strategies include alterations of their lipopolysaccharides (LPSs), which have overall negative charges and are the initial targets of polymyxins (Moffatt et al., 2010). Such alterations can be achieved by covalent modifications of the lipid A moiety of LPS through the addition of phosphoethanolamine (PEtN) and 4-amino-4-deoxy-L-arabinose (L-Ara4N), deacylation, hydroxylation and palmitoylation by *pagP* (palmitoylation does not contribute to polymyxin resistance) (Ernst et al., 2001; Raetz et al., 2007). Other strategies include the utilization of an efflux pump and capsule formation (Campos et al., 2004; Padilla et al., 2010).

The most common LPS modification is the cationic substitution of the phosphate groups by L-Ara4N, which decreases the net negative charge of lipid A to 0, and the second most common is the PEtN modification, which decreases the net charge from −1.5 to −1 (Nikaido, 2003). The L-Ara4N modification is the most effective of the two modifications due to the nature of the charge alteration. The resultant net positive charge of the modified LPS reduces its binding to polymyxins, leading to resistance.

The activation of two-component systems (TCSs) involving PhoP/PhoQ and PmrA/PmrB is triggered by environmental stimuli and specific mutations within the TCSs that result in their constitutive activation and subsequent overexpression of LPS-modifying genes (Gunn and Miller, 1996; Gunn et al., 2000; Trent et al., 2001b; Abraham and Kwon, 2009; Barrow and Kwon, 2009; Miller et al., 2011). The activation of the PmrA/PmrB TCS leads to the upregulation of the *pmrCAB* and *arnBCADTEF*-*pmrE* (also called *pmrHFIJKLM-ugd*) operons that mediate the synthesis and transfer of PEtN and L-Ara4N, respectively, to lipid A (Gunn, 2001; Raetz et al., 2007; Yan et al., 2007). The PhoP/PhoQ TCS is known to contribute to polymyxin resistance by indirectly activating the PmrA/PmrB TCS via PmrD, except in various bacteria, such as *E. coli* (Kox et al., 2000; Kato et al., 2003; Winfield and Groisman, 2004). Once activated, the phosphorylated PmrA binds to the promoter region of the *arnBCADTEF* operon, increasing the recognition and binding of RNA polymerase and resulting in the upregulation of the operon (Wosten and Groisman, 1999).

There have been reports summarizing the mechanisms of resistance to polymyxins (Nation and Li, 2009; Falagas et al., 2010; Lim et al., 2010; Loutet and Valvano, 2011; Biswas et al., 2012; Cai et al., 2012); however, the lack of updated and comprehensive reports on the different mechanisms mediating or contributing to polymixin resistance including among the intrinsically-resistant bacteria has further necessitated this work.

## Acquired resistance to polymyxins in enterobacteriaceae

### Role of LPS modifications

#### PmrA/PmrB and PhoP/PhoQ two-component system-mediated LPS modifications

**Figure 1:**
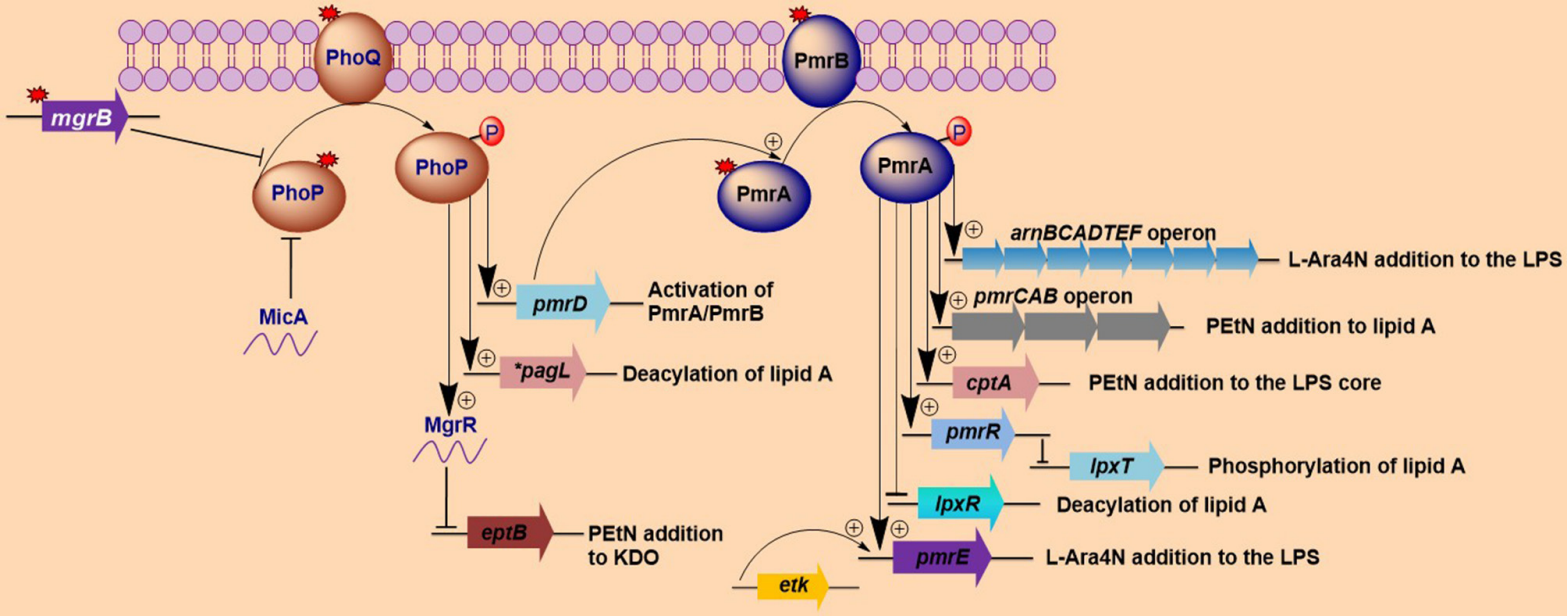
**Activation of lipopolysaccharide-modifying genes involved in polymyxin resistance in Gram-negative bacteria**. Both MgrB and MicA (in *Escherichia coli*) exert negative feedback on the *phoP/phoQ* regulatory system, while mutations (denoted by red-colored star symbols) in *mgrB* or *phoP/phoQ* typically lead to the constitutive induction of the *phoP/phoQ* two-component system. The activation of this two-component system (*phoP/phoQ*) activates *pagL* (which deacylates lipid A in *Salmonella*) and *pmrD* (which in turn activates *pmrA*) and represses *eptB* via the activation of MgrR, with the resultant lipopolysaccharide (LPS) modifications all participating in the mediation of polymyxin resistance. Additionally, the *phoP/phoQ* regulatory system can directly activates the *arnBCADTEF*operon in some bacteria such as *Klebsiella pneumoniae*. The repression of *eptB* prevents the modification of 3-deoxy-D-manno-oct-2-ulosonic acid (Kdo) with phosphoethanolamine (PEtN). The *pmrA/pmrB* two-component system is activated via *pmrD* (which is activated by *phoP*) or through mutations in the *pmrA/pmrB* genes. Once induced, the phosphorylated *pmrA* activates the *arnBCADTEF* and *pmrE* genes, which collectively modify LPSs with 4-amino-4-deoxy-L-arabinose (L-Ara4N). The lipid A and the LPS core are further modified with PEtN by the *pmrCAB* operon and *cptA*, respectively. Additional *pmrA*-activated genes include *pmrR*, which represses *lpxT* (that phosphorylates lipid A) upon activation and *lpxR* gene (which deacylates lipid A). Lastly, *etK* can additionally phosphorylate the *pmrE* gene. The findings illustrated here are limited to modifications that have been shown to affect sensitivity to polymyxins. ^*^pagL has only been found in *Salmonella*.

**Figure 2:**
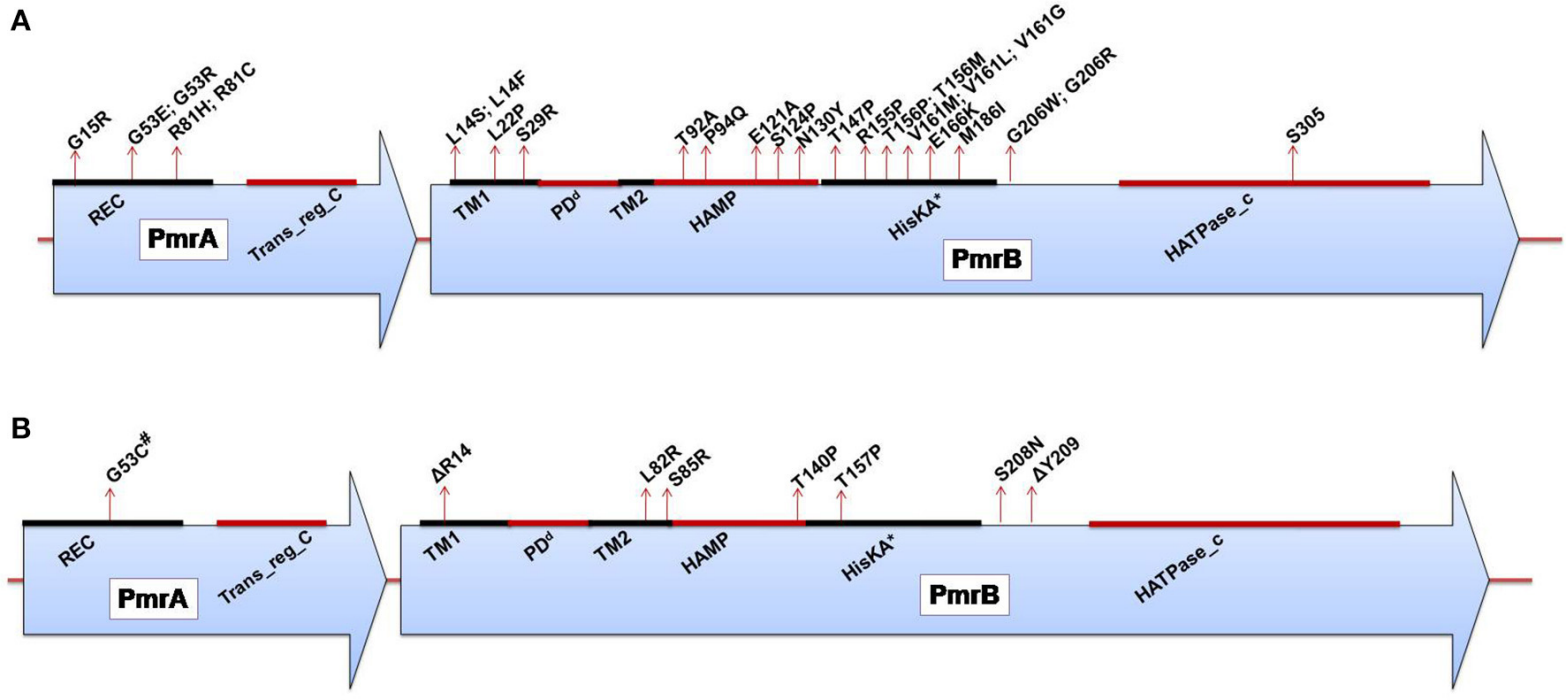
**(A)** Domains of the PmrA/PmrB two-component system and positions of all mutations conferring polymyxin resistance to *Salmonella enterica* serovar Typhimurium. *PmrA domains*, cheY-homologous receiver domain [REC]; aa 1–112. Transcriptional regulatory protein, C-terminal domain [Trans_reg_C]; aa 145–216. *PmrB domains*, First transmembrane domain [TM1]; aa 13–35. ^d^Periplasmic domain [PD]; aa 35–66. Second transmembrane domain [TM2]; aa 66–88. Histidine kinases, adenylyl cyclases, methyl-binding proteins, and phosphatases [HAMP domain]; aa 89–141. Histidine kinase A (phosphoacceptor) domain [HisKA]; aa 142–202. Histidine kinase-like ATPases [HATPase_c]; aa 249-356. ^*^HisKA, with the active site at H148 in PmrB of *Salmonella enterica* subsp. enterica serovar Typhimurium str. LT2 (GenBank accession no. AE006468). ^d^Periplasmic domain was not predicted in SMART but was assumed to be between the TM1 and TM2; aa, amino acid. **(B)** Domains of the PmrA/PmrB two-component system and positions of all mutations conferring polymyxin resistance to *Klebsiella pneumoniae*. *PmrA domains*, cheY-homologous receiver domain [REC]; aa 1–112. Transcriptional regulatory protein, C-terminal domain [Trans_reg_C]; aa 145–216.*PmrB domains*, First transmembrane domain [TM1]; aa 13–35. ^d^Periplasmic domain [PD]; aa 35–67. Second transmembrane domain [TM2]; aa 67–89. Histidine kinases, adenylyl cyclases, methyl-binding proteins, and phosphatases [HAMP domain]; aa 90–142. Histidine kinase A (phosphoacceptor) domain [HisKA]; aa 143–203. Histidine kinase-like ATPases [HATPase_c]; aa 250–358. ^*^HisKA, with the active site at H153 in PmrB of *K. pneumoniae* subsp. *pneumoniae* MGH 78578 (GenBank accession no. CP000647). ^#^Same mutation as that reported for colistin-resistant *Enterobacter aerogenes*. ^d^Periplasmic domain was not predicted in SMART but was assumed to be between TM1 and TM2; aa, amino acid; Δ, deletion.

***Salmonella enterica***. *Salmonella enterica* serovar Typhimurium (*S*. Typhimurium) has been used as a model bacterium for most studies aimed at elucidating the mechanisms of resistance to cationic antimicrobial peptides (CAMPs), including polymyxins. Vaara et al. have observed that the LPSs of a polymyxin-resistant *pmrA* mutant of an *S*. Typhimurium strain contains 4–6 times more L-Ara4N in lipid A than the parent strain in addition to an increased level of PEtN (Vaara et al., 1981; Trent et al., 2001b). In polymyxin-resistant *Salmonella*, one principal mechanism underlying this modification, apart from external stimuli such as Mg^2+^, involves mutations in the PmrA/PmrB and PhoP/PhoQ TCSs. Mutations in these systems can cause their constitutive overexpression, resulting in the activation of the *arnBCADTEF* and *pmrCAB* operons and the modification of lipid A by L-Ara4N and PEtN, respectively (Figure 1) (Roland et al., 1993; Guo et al., 1997; Gunn et al., 1998; Zhou et al., 2001; Lee et al., 2004). Several mutations that activate the PmrA/PmrB TCS have been characterized in *in vitro*-selected mutant polymyxin-resistant *S*. Typhimurium (Table 1). Roland et al. have reported a missense mutation in *pmrA*, resulting in an amino acid substitution of R81H (Roland et al., 1993). An extensive genetic analysis of *pmrA*/*pmrB* of spontaneous colistin-resistant mutants has further identified numerous mutations, including a total of 27 independent missense mutations in the *pmrA* and *pmrB* genes that all result in the elevated expression of *arnB* (*pmrH*) (Sun et al., 2009). The minimum inhibitory concentrations (MICs) of colistin for the spontaneous mutants reported range from 0.25 to 4.4 mg/L, with most mutants displaying a 20–30-fold MIC increase. For PmrA, all mutations occurred in the phosphate receiver domain, while mutations occurred in 4 out of the 6 predicted domains in PmrB (Figure 2A). The histidine kinase gene *pmrB* seems to be the more common site for bacterial mutations compared to the response regulator gene *pmrA*. However, not all missense mutations in *pmrA*/*pmrB* result in their constitutive activation and polymyxin resistance. Some non-polymyxin resistance-causing missense mutations have been observed in the *pmrA*/*pmrB* of various *Salmonella* serotypes (Agerso et al., 2012).

**Table 1:** **Mutations in two-component systems that result in their constitutive activations**.

**Bacteria**	**Gene**	**Mutation in aa**	**References**
*Salmonella enterica*	*pmrA*	R81H, R81C	Roland et al., 1993; Sun et al., 2009
		G15R	Sun et al., 2009
		G53E, G53R	
	*pmrB*	L14S, L14F	
		L22P	
		S29R	
		T92A	
		P94Q	
		E121A	
		S124P	
		N130Y	
		T147P	
		R155P	
		T156P, T156 M	
		V161M, V161L, V161G	
		E166K	
		M186I	
		G206W, G206R	
		S305R	
*Klebsiella pneumoniae*	*pmrA*	G53C	Olaitan et al., 2014b
	*pmrB*	L82R	Cannatelli et al., 2014b
		T157P	Jayol et al., 2014
		S85R	Olaitan et al., 2014b
		T140P	
		ΔR14	Choi and Ko, 2014
		ΔY209	
		T157P	
		S208N	
*Enterobacter aerogenes*	*pmrA*	G53C	Diene et al., 2013
*Acinetobacter baumannii*	*pmrA*	M12I	Arroyo et al., 2011
		S119T	
		E8D	Lesho et al., 2013; Rolain et al., 2013
		P102H	Adams et al., 2009
	*pmrB*	T13N	
		A227V	
		P233S, P233T	
		A262P	
		I121F	Park et al., 2011
		A183T	
		A184V	
		P190S	
		T192I	
		Q228P	
		S14L	Beceiro et al., 2011
		L87F	
		M145K	
		A227V	
		P233S	
		N353Y	
		F387Y	
		S403F	
		P170L	Pournaras et al., 2014
		P233S	
		ΔA32-E35	Arroyo et al., 2011
		D64V	
		A80V	
		ΔL160	
		P170Q, P170L	
		L208F	
		A226V	
		R231L	
		P233S	
		T235I	
		N256I	
		R263P, R263C	
		Q277H	
		G315D	
		P377L	
		S17R	Lesho et al., 2013
		Y116H	
		T232I	
		R263L	
		A227V	Kim et al., 2014b
		P233S	
		*Fr*F26	
*Pseudomonas aeruginosa*	*pmrA*	L157Q	Lee and Ko, 2014
	*pmrB*	M292T	Abraham and Kwon, 2009
		L243Q	Moskowitz et al., 2004
		A248V	
		ΔD45	Schurek et al., 2009; Moskowitz et al., 2012
		L14P	Moskowitz et al., 2012
		A54V	
		R57H	
		R79H	
		R135Q	
		G188D	
		A248T	
		S257N	
		R259H	
		M292I	
		P456S	
		V15I	Lee and Ko, 2014
		M48L	
		A67T	
		D70N	
		L167P	
		H340R	
		T343A	
		A247T	Owusu-Anim and Kwon, 2012
		M292T	
		Y345H	
		V281I	Choi and Ko, 2014
		F237L	
*K. pneumoniae*	*phoP*	G385S	Olaitan et al., 2014b
		L26Q	
	*phoQ*	L96P	
		L348Q	
		S174N	Choi and Ko, 2014
*P. aeruginosa*	*phoQ*	V260G	Owusu-Anim and Kwon, 2012
		H223R	
		V152 trunc.	
		A143V	Lee and Ko, 2014
		K123Q	
		*Fr* I421-428,X 429	Miller et al., 2011
		I421X^d^	
		D433X^d^	
		ΔL364-G365	
		ΔL364-G365, R444C	
		R6C, ΔL364-G365	
		ΔV57-Q332	
		K123E	Choi and Ko, 2014
		R214H	
		V184G	
		Q133E	
		A207R	
		N104I	
	*parR*	N24S	Choi and Ko, 2014
		L18I	
		S24N	
		M59I	Muller et al., 2011
		E156K	
	*parS*	L14Q	
		V101M	
		L137P	
		Q232E	Choi and Ko, 2014
		G361R	
		V295L^†^	Lee et al., 2014a
		A296P^†^	
		H398R	
	*colR*	D32N	Muller et al., 2011
	*colS*	A106V	Gutu et al., 2013
	*cprS*	R241C	

aa, amino acid; Δ, deletion; trunc., truncation; Fr, frameshift mutation; X^d^, premature termination of amino acid;

† *predicted not to affect protein's function according to SIFT (Sorting Intolerant From Tolerant) analysis*.

It has been observed that up to 88% of the 4′-phosphate of lipid A can be extensively modified with L-Ara4N in *pmrA* mutants of *S*. Typhimurium (Helander et al., 1994). Generally, L-Ara4N modifies the 4′-phosphate, while PEtN modifies the 1-phosphate; however, in some cases, either one or two L-Ara4N and/or PEtN can be added to the 4′-phosphate and 1-phosphate of lipid A (Zhou et al., 2001; Lee et al., 2004). These two modifications are mediated by the *arnBCADTEF* operon and *pmrC* (also called *eptA*), respectively. Additionally, the phosphorylated heptose-I residue (LPS core) can be further modified with PEtN by the *cptA* gene (Figure 1) (Tamayo et al., 2005a). *arnBCADTEF* (also referred to as the *pmrF* or *arnT* operon), *pmrC* and *cptA* are all regulated by *pmrA* in *Salmonella* (Tamayo et al., 2005b). The cationic modifications of these phosphate groups in LPSs are responsible for their reduced binding to polymyxins.

With regard to polymyxin resistance, the modification of LPSs by L-Ara4N confers a higher level of resistance than PEtN modifications (Tamayo et al., 2005a). Therefore, in terms of LPS modification-mediated polymyxin resistance in *S*. Typhimurium, the *arnT* operon is the most important, followed by *pmrC* and *cptA*.

Furthermore, the 1-phosphate of lipid A can be phosphorylated to form 1-diphosphate (1-PP) by LpxT (Jones et al., 2008; Touze et al., 2008). This alteration increases the net negative charge of lipid A and reduces the efficiency of the PEtN modification (Herrera et al., 2010). These characteristic processes can increase the overall sensitivity of *S*. Typhimurium to polymyxins. However, LpxT activity is inhibited by the activation of *pmrA* (Herrera et al., 2010) via PmrR (Pmr regulator) in *S*. Typhimurium, which further enhances resistance to polymyxins (Figure 1) (Kato et al., 2012). The biology of these genes (*lpxT* and *pmrR*) with respect to polymyxin resistance has yet to be fully elucidated, but it is highly probable that they may have a direct impact on this resistance considering their involvement in altering the overall charges of LPSs.

In *Salmonella*, the *R*-3-hydroxymyristate at position 3 of lipid A can be removed (deacylated) by *pagL*, which itself is activated by PhoP (Trent et al., 2001a). PagL is normally latent due to its inhibition by L-Ara4N and PEtN modifications of lipid A (Kawasaki et al., 2005, 2007), but the PagL-mediated deacylation of lipid A can occur in strains that are unable to modify this lipid A with either L-Ara4N or PEtN. Consequently, in such strains, the PagL-dependent deacylation of this lipid A increases polymyxin resistance (Kawasaki et al., 2007). This finding implies that the various forms of lipid A modifications, particularly L-Ara4N and PEtN modifications and deacylation, may be compensatory to each other in certain bacteria, such as *Salmonella*. This relationship further depicts the complexity and intricacy of the interactions among the various mechanisms that mediate bacterial resistance to antimicrobial peptides, including polymyxins.

Some genes that have been implicated to be involved in polymyxin resistance and are independent of the PmrA/PmrB and PhoP/PhoQ genes include *rpoN*, which is an alternative sigma factor known to activate the transcription of several genes (Kazmierczak et al., 2005). *S*. Typhimurium with inactivated *rpoN* displayed polymyxin resistance by about two-fold, independent of *pmrA*, possibly due to the downregulation of polymyxin resistance-related gene(s) that are *rpoN*-regulated (Barchiesi et al., 2009).

The clinical relevance of polymyxin resistance in *Salmonella* currently seems to be non-existent, which is possibly because polymyxins are not currently used for treating infections caused by this bacterium. However, *in vivo* colistin resistance has been observed in animals such as pigs and poultry birds (EMEA, 2002; de et al., 2012; Morales et al., 2012; Kempf et al., 2013; Quesada et al., 2014), and such strains can be horizontally transmitted to humans.

***Klebsiella pneumoniae***. There have been a considerable number of studies aiming to elucidate polymyxin resistance in *K. pneumoniae*. In a genetically uncharacterized polymyxin-resistant strain of *K. pneumoniae*, the phosphate groups of lipid A have been observed to contain five times more L-Ara4N than the susceptible strain (Helander et al., 1996). This altered outer membrane composition is known to lower the negative charge of the outer membrane of *K. pneumoniae* (Velkov et al., 2013a), leading to the reduced interaction of this membrane with polymyxins. A molecular characterization of the structural alterations of LPSs in *K. pneumoniae* with regard to polymyxin resistance has similarly shown the involvement of *phoP/phoQ* and *pmrA/pmrB* (Cheng et al., 2010). It has been observed that the *phoP/phoQ* and *pmrA/pmrB* systems are upregulated in *K. pneumoniae* exposed to polymyxins (Kim et al., 2014a), indicating that these systems are involved in polymyxin resistance in this bacterium. The constitutive activation of the *pmrA/pmrB* system can also be caused by missense mutations in *pmrA* or *pmrB*, leading to the subsequent upregulation of *pmrC* and the *arnBCADTEF* operon, resulting in the synthesis and addition of PEtN and L-Ara4N, respectively, to lipid A, as shown in Figure 1. Recently, various occurrences of such mutations have been identified in both the *pmrA* and *pmrB* genes of clinical and non-clinical isolates of colistin-resistant *K. pneumoniae*, as shown in Table 1 and Figure 2B (Cannatelli et al., 2014b; Jayol et al., 2014; Olaitan et al., 2014b). In all the studies, the MIC range of colistin has been reported to be 3–16 mg/L as shown by an Etest. Interestingly, similar mutations in the same loci have also been observed in colistin-resistant *Salmonella* and *Enterobacter aerogenes* (with a colistin MIC of 32 mg/L) (Sun et al., 2009; Diene et al., 2013). However, several synonymous mutations that are not responsible for resistance have also been observed in *pmrA* and *pmrB*. Similarly, possible mutations in *phoQ* genes that result in resistance have also been observed in colistin-resistant *K. pneumoniae* (Olaitan et al., 2014b).

One profound molecular mechanism that leads to the emergence of colistin resistance in *K. pneumoniae* that has recently been discovered is the mutation/inactivation of the *mgrB* gene (Cannatelli et al., 2013), which is a conserved gene of 141 nucleotides in length (the length varies in naturally colistin-resistant Enterobacteriaceae) encoding a short, 47-amino acid transmembrane protein that exerts negative feedback on the PhoP/PhoQ regulatory system. It has been proposed that MgrB accomplishes this feedback by inhibiting the kinase activity of PhoQ and/or stimulating its phosphatase activity, which subsequently suppresses PhoP phosphorylation, leading to the repression of PhoP-regulated genes (Figure 1) (Lippa and Goulian, 2009). In accordance with this proposed mechanism, the deletion of *mgrB* in *E. coli* has been observed to result in the upregulation of PhoP-regulated genes (Lippa and Goulian, 2009).

**Figure 3:**
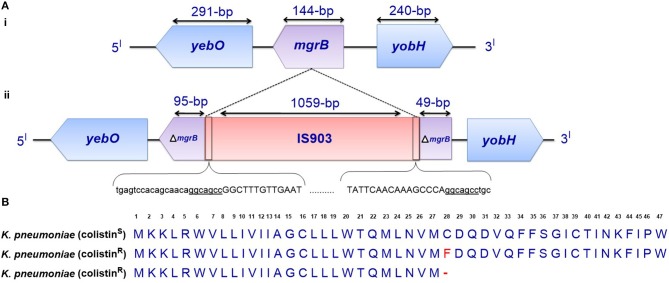
**(A)** Genetic representation of the *phoP/phoQ* negative regulator, *mgrB*. (i) *K. pneumoniae* with intact *mgrB* (colistin-susceptible), and (ii) *K. pneumoniae* with *mgrB* truncated by an insertion sequence (colistin-resistant). **(B)** Alignment of unmutated MgrB from colistin-susceptible *K. pneumoniae* and mutated MgrB from a colistin-resistant strain with a missense mutation and premature termination of MgrB. Premature termination (-).

Disruption of the *mgrB* gene in *K. pneumoniae* has been identified to play a prominent role in polymyxin resistance in this bacterium. Various disruptions in *mgrB* have recently been described in diverse clinical and non-clinical isolates of colistin-resistant *K. pneumoniae* and *K. oxytoca*, including insertional inactivation by an IS5-like element and other insertion sequences (Figure 3A) (Cannatelli et al., 2013; López-Camacho et al., 2013; Gaibani et al., 2014; Olaitan et al., 2014b; Poirel et al., 2014). Additional alterations that have been reported in *mgrB* include a non-sense mutation leading to the premature termination of the MgrB transmembrane protein and missense mutations resulting in amino acid substitutions (Figure 3B) (Cannatelli et al., 2014a; Olaitan et al., 2014b; Poirel et al., 2014). The range of MICs for colistin that has been reported in these isolates is 4-64 mg/L, as shown by Etest colistin strips. Several insertion sequences, such as those of the IS5-like, IS903B, IS1F-like and ISKpn14 elements belonging to several IS families (especially the IS5 family), have been observed to lead to the truncation of *mgrB*. An IS5D-like element initially localized on the plasmid has been observed to transposed to the chromosome and inactivate *mgrB* (Cannatelli et al., 2014a; Olaitan et al., 2014b). Likewise, several amino acid substitutions, such as C28F (which has already been reported to affect PhoQ activity in *E. coli* (Lippa and Goulian, 2012), have been described in colistin-resistant *K. pneumoniae* (Cannatelli et al., 2014a; Olaitan et al., 2014b). Furthermore, a small deletion or the complete deletion of the *mgrB* locus has been reported in various colistin-resistant strains (Cannatelli et al., 2014a; Olaitan et al., 2014b). Complementation studies carried out on some of these *mgrB* mutant strains have resulted in the restoration of colistin sensitivity (Cannatelli et al., 2013, 2014a). This disruption of *mgrB* can result in the upregulation of the *arnBCADTEF* operon, which adds L-Ara4N to lipid A (Cannatelli et al., 2013). The upregulation of the *arnBCADTEF* operon is attributed to phosphorylated PmrA, which is activated by PmrD, which is in turn activated by the phosphorylation of PhoP resulting from the disruption of MgrB (Cheng et al., 2010; Kim et al., 2014a). Moreover, it has been demonstrated that activated PhoP can also directly activate the *arnBCADTEF* operon in *K. pneumoniae* independent of the PmrD- and PmrA-activated proteins in a phenomenon termed the feedforward connector loop (FCL) (Mitrophanov et al., 2008). We have recently observed that the mutation/inactivation of *mgrB* accounts for greater colistin resistance among resistant *K. pneumoniae* compared with that resulting from TCS mutations (*pmrA*/*pmrB* or *phoP/phoQ*) (Olaitan et al., 2014b). This observation demonstrates that *mgrB* plays an important role in polymyxin resistance in *K. pneumoniae*.

A cross-regulatory interaction between PhoP/PhoQ and the regulator of the capsule synthesis (Rcs) phosphorelay system has been demonstrated in *K. pneumoniae*. Mutant *rcsB* upregulates the expression of *phoP/phoQ*, implying that the Rcs system normally downregulates the *phoP/phoQ* system (Llobet et al., 2011). In light of this crosstalk between the two regulatory systems, it is plausible that a mutation in *rcsB* may play a role in polymyxin resistance via the upregulation of the *phoP/phoQ* system in *K. pneumoniae*, although this mechanism remains to be demonstrated in polymyxin-resistant isolates.

***Escherichia coli***. In polymyxin-resistant *E. coli*, lipid A is typically modified with 2-aminoethanol and also with L-Ara4N (Nummila et al., 1995). The 2-aminoethanol molecule is added to the glycosidically linked diphosphate, whereas L-Ara4N is attached to the 4′-phosphate (Nummila et al., 1995). The importance of the L-Ara4N modification for polymyxin resistance in *E. coli* has been further demonstrated in other studies (Yan et al., 2007).

As depicted in Figure 1, in addition to the phosphorylation of Ugd (or PmrE) and the *arnT* operon by the phosphorylated PmrA from the PmrA/PmrB TCS (Aguirre et al., 2000), Etk (a tyrosine-kinase) has been observed to phosphorylate Ugd, thereby participating in polymyxin resistance in *E. coli* (Lacour et al., 2006); the mutant *E. coli* strain displayed a reduced survival rate in the presence of polymyxin B. The phosphorylation of the Ugd protein (a UDP-glucose dehydrogenase) by Etk increases Ugd dehydrogenase activity. This elevated activity leads to an increase in the synthesis of UDP-glucuronic acid, which is the starting material for L-Ara4N synthesis by the *arnT* operon (Lacour et al., 2008). Interestingly, *etk* is expressed under conditions also known to activate the PmrA/PmrB system (Lacour et al., 2008). This finding indicates that, apart from the PmrA/PmrB system, polymyxin resistance via the modification of LPSs with L-Ara4N may be partially regulated by *etk*. Some studies have reported that Ugd is not directly activated by PmrA (Wosten and Groisman, 1999), but others have provided contrasting results (Aguirre et al., 2000). However, it is possible that *etk* is a principal activator of Ugd.

The 1-phosphate group of lipid A can be further modified by PEtN, albeit to a lesser extent (Kim et al., 2006). The addition of PEtN is mediated by *pmrC* (also called *eptA*) and regulated by PmrA (Raetz et al., 2007). The lipid A of *E. coli* O157:H7 has been found to be distinctly modified by PEtN, most likely due to the increased activity of *pmrC* in this serotype. This modification confers slight resistance to the cationic antimicrobial peptide PMBN (Kim et al., 2006). These findings demonstrate that alterations with L-Ara4N confer increased resistance to polymyxins compared with that of PEtN.

*mgrR* is another genetic determinant that has been shown to mediate polymyxin resistance in *E. coli*. It is an Hfq-dependent sRNA that is one of the regulons known to be regulated by the PhoP/PhoQ regulatory system in *E. coli*. *mgrR* is conserved in other enteric bacterial genera, such as *Salmonella*, *Citrobacter*, *Enterobacter*, and *Klebsiella*. It has been described to negatively regulate *eptB*, which mediates the modification of the outer Kdo (3-deoxy-D-*manno*-octulosonic acid) residues of LPSs with PEtN (Figure 1) (Reynolds et al., 2005; Raetz et al., 2007). *eptB* is indirectly influenced by the PhoP/PhoQ system (Moon and Gottesman, 2009). The modification of Kdo with PEtN reduces the net negative charges of LPSs, eventually leading to polymyxin resistance (Moon and Gottesman, 2009). It has been observed that the deletion of *mgrR* in *E. coli* increases resistance to polymyxin B (Moon and Gottesman, 2009) as a result of the increased PEtN modification of Kdo. The expression of the PhoP/PhoQ TCS itself has been observed to be regulated by an Hfq-dependent sRNA termed MicA in *E. coli* and has been shown to repress the expression of PhoP (Coornaert et al., 2010). This process controls several PhoP-regulated genes, including MgrR. Thus, it is possible that MicA can also influence polymyxin resistance, although this influence has yet to be demonstrated.

It has recently been reported that cross-talk between QseB/QseC (quorum-sensing regulatory proteins) and PmrA/PmrB exists in *E. coli*. PmrB activates QseB in *E. coli* lacking QseC, and PmrA also directly regulates the transcription of QseB/QseC (Guckes et al., 2013). This interaction raises the possibility of cross-talk among other TCSs, which may influence the PhoP/PhoQ and/or PmrA/PmrB TCSs with respect to bacterial resistance to cationic antimicrobial peptides.

#### Underacylation of lipid a and its effect on polymyxin resistance

***Salmonella enterica***. The importance of the myristoylation of lipid A (a late secondary acylation) with regard to polymyxin resistance has been highlighted in *Salmonella*. *Salmonella msbB* strains (*lpxM* mutants) that produce predominantly penta-acylated lipid A (underacylated) and are unable to add the myristoyl group to this lipid A display sensitivity to polymyxin (Tran et al., 2005; Murray et al., 2007). This sensitivity occurs because the myristoylation of lipid A is essential for the L-Ara4N modification of the phosphate groups of lipid A (Tran et al., 2005). Nonetheless, a mutation in *pmrA* can still confer polymyxin resistance to a *Salmonella lpxM* mutant as a result of an alternative lipid A modification, such as that involving PEtN, although this resistance is lower than that conferred by the addition of L-Ara4N. Therefore, the secondary acylation of lipid A is important for the addition of L-Ara4N (but not PEtN) to the phosphate groups of this lipid and for resistance to polymyxins.

***Klebsiella pneumoniae***. In *K. pneumoniae*, the *lpxM* gene also encodes the enzyme involved in the addition of the myristoyl group to lipid A, which results in the formation of hexa-acylated lipid A. An *lpxM* mutant of *K. pneumoniae* that produces predominantly penta-acylated lipid A has been found to be 8- to 16-fold more sensitive to both polymyxin B and colistin than the wild type with hexa-acylated lipid A (Clements et al., 2007). Velkov et al. have shown that the LPSs of the *K. pneumoniae* lpxM mutant strain with a colistin MIC of 0.25 mg/L display a four-fold higher binding affinity to polymyxins compared to the wild-type strain, with an MIC of 4 mg/L (Velkov et al., 2013b).

Similarly, a greater degree of acylation has been observed in polymyxin-resistant *K. pneumoniae* by Helander et al. (1996). Similar to *Salmonella*, underacylation has been reported to play an important role in polymyxin resistance in *K. pneumoniae*, likely due to the inability of underacylated *K. pneumoniae* to perform the L-Ara4N modification of lipid A, as has been noted in other enteric bacteria (Tran et al., 2005). It has been alternatively proposed that the increased susceptibility of underacylated *K. pneumoniae*, which results in fewer acyl chains, leads to the enhanced insertion of the polar heads or the fatty acid tails of polymyxins into membranes and thus a higher binding affinity of lipid A to these antibiotics (Clements et al., 2007; Velkov et al., 2013b).

***Escherichia coli***. The myristoylation of lipid A by *lpxM* resulting in a hexa-acylated lipid A is likewise important for the addition of L-Ara4N to the lipid A of *E. coli*. Therefore, a loss of lipid A myristoylation in an *lpxM* mutant results in a lack of L-Ara4N modification and a subsequent decrease in polymyxin resistance (Tran et al., 2005).

#### Other LPS modifications mediating polymyxin resistance

The removal of the 3′-acyloxyacyl residue from lipid A has been observed in *S*. Typhimurium. This removal is mediated by *lpxR*, which is a hydrolase gene that encodes 3′-*O*-deacylase and has been shown to be present in other Gram-negative bacteria. *lpxR* is usually latent because its expression is inhibited by PmrA/PmrB-regulated lipid A modifications (Reynolds et al., 2006). The removal of 3′-acyloxyacyl results in the attachment of a reduced number of acyl groups to lipid A, and this alteration has been demonstrated to contribute to polymyxin resistance (Helander et al., 1996; Tran et al., 2005; Clements et al., 2007; Murray et al., 2007). The activation of *lpxR* may play a role in polymyxin resistance, but the contribution of *lpxR* to this resistance has not yet been demonstrated.

Recently, genomic analysis of colistin-resistant *K. pneumoniae* isolates has revealed that these isolates harbor eight distinct mutations compared to susceptible isolates that have been found in both non-coding and coding regions. Mutations have been detected in genes encoding a microcin transporter, a putative membrane protein, a putative transport protein and the methyl viologen-resistance protein SmvA (Snitkin et al., 2012), and the isolates display a colistin MIC range of 4–128 mg/L. Another study involving a comparative genomic analysis of two colistin-resistant *K. pneumoniae* strains identified non-synonymous mutations in the *waaL*, *rfbA*, and *vacJ* genes. All three of these genes are known to be involved in the biosynthesis of outer membrane proteins (Sassera et al., 2013). However, it is not yet known if these genes or their mutations contribute to colistin resistance. Furthermore, *Salmonella enterica* with a mutation in *waaP* (a gene encoding a protein involved in the phosphorylation of the heptose residue of the LPS inner core) has been shown to display a higher susceptibility to polymyxins (Yethon et al., 2000).

Overall, the activities of most of these genes are outer membrane-related, and any gene that acts to undermine the integrity of the bacterial outer membrane may play a role in polymyxin resistance (Snitkin et al., 2012). Therefore, some of these aforementioned genes need to be further scrutinized.

### Role of capsule in polymyxin resistance

It has been reported that *K. pneumoniae* is able to shed capsular polysaccharides (CPSs) from its surface. The released CPSs are able to trap or bind to polymyxins, thereby reducing the quantity of drug that reaches the bacterial cell surface, leading to increased polymyxin resistance (Llobet et al., 2008). The underlying mechanism is attributed to electrostatic interactions between the cationic polymyxins and anionic CPSs.

It has been further observed that CPSs play a direct role in resistance to antimicrobial peptides, including polymyxin B, by reducing their interactions with the bacterial outer membrane, mainly through the upregulation of capsular biosynthesis genes. CPS genes are further dependent on a certain CPS biosynthesis threshold for resistance (Campos et al., 2004). However, the role of the capsule in mediating polymyxin resistance remains under debate. Various studies have reported that the capsule does not contribute to resistance against antimicrobial peptides, including polymyxin B (Weiss et al., 1982; Clements et al., 2007). Interestingly, the *ugd* gene plays a dual role in CPS and L-Ara4N biosyntheses (Lacour et al., 2006), and its phosphorylation serves as a bridge between capsular synthesis and polymyxin resistance (Lacour et al., 2008).

### Role of efflux pump in polymyxin resistance

A few studies have shown that efflux pumps can also contribute to polymyxin resistance. The efflux pumps that have been studied include AcrAB and KpnEF. It has been observed that *K. pneumoniae* with a mutation in *acrB* is significantly more susceptible to polymyxin B than the wild-type strain and that polymyxin B is pumped out of *K. pneumoniae* in an energy-dependent manner (Padilla et al., 2010). Another study has reported that mutant KpnEF (a member of the small MDR efflux pump family) is more susceptible to several antibiotics, including colistin, compared with wild-type *K. pneumoniae* and that this mutant displays a two-fold reduction in the colistin MIC compared with the wild-type strain (Srinivasan and Rajamohan, 2013). Interestingly, the same *kpnEF* mutant also displays an impairment in capsular synthesis (Srinivasan and Rajamohan, 2013).

## Acquired resistance to polymyxins in non-fermentative bacilli

### Acinetobacter baumannii

Two primary mechanisms that provide colistin resistance have been described in *A. baumannii* to date. The first is the modification of the lipid A moiety of LPS with PEtN as a result of mutations in the *pmrA*/*pmrB* TCS. The second is the complete loss of LPSs caused by either mutations or the insertional inactivation of lipid A biosynthesis genes (Moffatt et al., 2010, 2011).

#### PmrCAB-mediated LPS modifications

**Figure 4:**
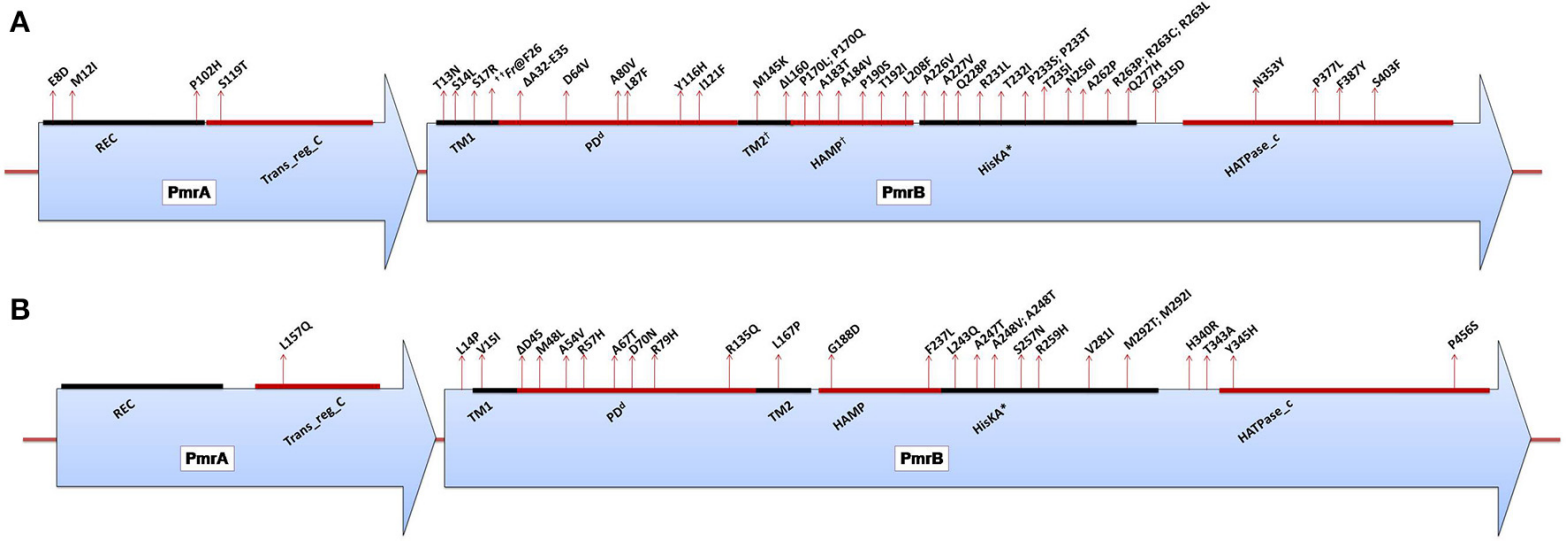
**(A)** Domains of the PmrA/PmrB two-component system and positions of all mutations conferring polymyxin resistance to *Acinetobacter baumannii*. *PmrA domains*, cheY-homologous receiver domain [REC]; aa 2–112. Transcriptional regulatory protein, C-terminal domain [Trans_reg_C]; aa 150–221. *PmrB domains*, First transmembrane domain [TM1]; aa 10–29. ^d^Periplasmic domain [PD]; aa 29–142. ^†^Second transmembrane domain [TM2]; aa 142–164. ^†^Histidine kinases, adenylyl cyclases, methyl-binding proteins, and phosphatases [HAMP domain]; aa 145–214. Histidine kinase A (phosphoacceptor) domain [HisKA]; aa 218–280. Histidine kinase-like ATPases [HATPase_c]; aa 326–437. ^*^HisKA, with the active site at H228 in PmrB of *Acinetobacter baumannii* ATCC17978 (GenBank accession no. CP000521). ^d^Periplasmic domain was not predicted in SMART but was assumed to be between TM1 and TM2. ^†^TM2 and HAMP overlapped based on SMART prediction. ^††^*Fr* denotes frameshift mutation; aa, amino acid. **(B)** Domains of the PmrA/PmrB two-component system and positions of all mutations conferring polymyxin resistance to *Pseudomonas aeruginosa. PmrA domains*, cheY-homologous receiver domain [REC]; aa 1–112. Transcriptional regulatory protein, C-terminal domain [Trans_reg_C]; aa 145–216. *PmrB domains*, First transmembrane domain [TM1]; aa 15–37. ^d^Periplasmic domain [PD]; aa 38–160. Second transmembrane domain [TM2]; aa 161–183. Histidine kinases, adenylyl cyclases, methyl-binding proteins, and phosphatases [HAMP domain]; aa 186–238. Histidine kinase A (phosphoacceptor) domain [HisKA]; aa 239–304. Histidine kinase-like ATPases [HATPase_c]; aa 348–459. ^*^HisKA, with the active site at H249 in PmrB of *Pseudomonas aeruginosa* PAO1 (GenBank accession no. AE004091). ^d^Periplasmic domain was not predicted in SMART but was assumed to be between TM1 and TM2; aa, amino acid.

*A. baumannii* does not possess the genetic machinery required for L-Ara4N biosynthesis; however, it contains an ortholog of the *pmrCAB* operon that mediates the addition of PEtN to its lipid A (Adams et al., 2009). Similar to enteric bacteria, the PmrA/PmrB TCS has been shown to mediate resistance to colistin in this bacterium. Mutations in *pmrA* and/or *pmrB* have been widely observed to induce the constitutive expression of *pmrA* (the cognate response regulator) and the subsequent autoregulation of the promoter region of the *pmrCAB* operon, which leads to the modification of the 4′-phosphate or 1-phosphate group of lipid A with PEtN. Missense or small indel mutations in the *pmr* locus (primarily in *pmrB*) in both colistin-resistant clinical isolates (*in vivo*) and *in vitro*-selected isolates have been shown to result in colistin resistance in *A. baumannii* (Table 1) (Arroyo et al., 2011; Beceiro et al., 2011; Lesho et al., 2013; Rolain et al., 2013; Snitkin et al., 2013). Mutations have been reported in different domains of both PmrA and PmrB (Figure 4A) (Arroyo et al., 2011; Beceiro et al., 2011; Lesho et al., 2013; Rolain et al., 2013). Additionally, *pmrB* appears to be the most commonly mutated gene in *A. baumannii*, similar to enteric bacteria (Figure 4A). In most of these studies, the MICs of colistin have ranged from 4 to 256 mg/L and from 4 to 128 mg/L as shown by Etest and broth microdilution techniques, respectively.

The reversion from a resistant to a susceptible phenotype has been observed in some strains in which the mutant *pmr* strain acquires an additional mutation (a compensatory mutation) elsewhere in the *pmr* locus that counteracts the hyper-activation of the TCS caused by the first mutation (Adams et al., 2009; Snitkin et al., 2013). It is also possible that the mutant *pmr* gene may revert back to its unmutated form, thereby reversing the resistance phenotype (Snitkin et al., 2013). However, compensatory mutations have not been observed in all revertants, with some strains maintaining the mutation in *pmrB*. This finding indicates the possible involvement of other unknown gene(s) in addition to the *pmrA/pmrB* locus in the mediation of colistin resistance and its reversion in some colistin-resistant strains. This idea is supported by the findings of Park et al., who did not detect any mutations in the *pmrA/pmrB* of six colistin-resistant *A. baumannii* clones out of 30 resistant isolates analyzed (Park et al., 2011), indicating the existence of other possible candidate gene(s). The identification of these genes is crucial for improving current knowledge of polymyxin resistance.

Colistin resistance in *A. baumannii* can emerge independently in a susceptible strain, and some strains may have a higher tendency of becoming resistant (Lesho et al., 2013). In some cases, *A. baumannii* possesses an auxiliary *pmrC*-like gene (named *eptA*), and some strains can have more than one copy of this gene (Arroyo et al., 2011; Lesho et al., 2013). It has been shown that the expression of this additional *pmrC*-like gene markedly increases in colistin-resistant strains. Interestingly, it has also been found to be located closer to mobile elements (prophage or integrase), suggesting the possibility of its horizontal acquisition (Lesho et al., 2013). The role of this auxiliary phosphoethanolamine transferase-encoding gene in colistin resistance in *A. baumannii* is still unknown.

***Other modifications***. Phosphorylation with PEtN usually occurs at the 4′-phosphate or 1-phosphate group of lipid A in *A. baumannii* colistin-resistant strains. In addition to this modification, a unique glycosylation of lipid A with galactosamine (hexosamine) at the 1-phosphate group has been recently reported in resistant strains (Pelletier et al., 2013). This unique modification is similar to the L-Ara4N modification in colistin-resistant enteric bacteria. Clinical isolates with both PEtN and galactosamine modifications have been reported, displaying colistin MICs of 1.5–48 mg/L as shown by a colistin Etest (Pelletier et al., 2013). The genetic basis underlying this modification remains to be determined. Furthermore, a predominance of hepta-acylated lipid A has been observed in colistin-resistant *A. baumannii* by Beceiro et al. (2011). This increase in lipid A acylation appears to be significant to colistin resistance, and the unique pattern of modifications (phosphorylation and glycosylation) observed in colistin-resistant *A. baumannii* is similar to that reported in enteric bacteria (Tran et al., 2005).

#### Loss of LPS-mediated colistin resistance

In colistin-resistant *A. baumannii*, mutations have been reported that involve a nucleotide substitution or deletion and an insertional inactivation with the insertion of ISAba11 element in the first three lipid A biosynthesis genes, namely *lpxA*, *lpxC*, and *lpxD*, resulting in the complete loss of LPSs (Moffatt et al., 2010, 2011). Resistant strains harboring such mutations display a >128 mg/L colistin MIC with broth microdilution, and they lack LPSs because of their inability to synthesize lipid A. Recent analysis of polymyxin B-resistant *A. baumannii* isolates has further shown the presence of unique mutations in the *lpxC* and *lpxD* genes (Lean et al., 2014) in addition to the mutations found in the *lpsB* gene encoding a glycosyltransferase (involved in the biosynthesis of the LPS core) that have also been implicated in colistin resistance in *A. baumannii* (Hood et al., 2013; Lean et al., 2014). Because lipid A is the initial target of colistin, its absence results in the loss of the colistin target site and a high level of resistance in *A. baumannii*.

Finally, transposon mutagenesis performed to elucidate the mechanisms underlying adaptive resistance to colistin has identified over 20 genes, most of which participate in processes that protect bacteria from osmotic stress, which likely occurs due to colistin exposure (Hood et al., 2013). Therefore, the expression of these genes stimulated by the syntheses of compatible solutes and the expression of proteases can attenuate such stress.

***Colistin heteroresistance in A. baumannii***. Colistin heteroresistance has been widely observed among *A. baumannii* clinical isolates at levels as high as 90% (Li et al., 2006; Barin et al., 2013). These resistant subpopulations have been reported to significantly contribute to both the regrowth and increased development of colistin resistance (Li et al., 2006). However, the underlying molecular mechanism involved in this phenomenon remains to be elucidated, and its understanding is critical due to the clinical significance of colistin heteroresistance.

***Fitness cost of colistin resistance in A. baumannii***. Development of colistin resistance in *A. baumannii* may incur fitness costs to this microorganism. Such costs have been observed in colistin-resistant isolates both *in vitro* and *in vivo* and include growth retardation, impaired virulence, and substantially reduced clinical invasiveness (Fernandez-Reyes et al., 2009; Lopez-Rojas et al., 2011; Rolain et al., 2011; Pournaras et al., 2014). Furthermore, Hraiech et al. have reported that colistin-resistant *A. baumannii* possesses a reduced ability to cause infections, including systemic dissemination and lung damage, in a rat model with pneumonia (Hraiech et al., 2013). Some of these biological costs, such as the impairment of virulence, have been linked with the reduced expression of metabolic proteins and of the OmpA porin (involved in the virulence of *Acinetobacter*) (Lopez-Rojas et al., 2011). Proteomic analysis of *in vitro*-selected colistin-resistant *A. baumannii* has revealed the differential expression of 35 proteins, the majority of which are downregulated in the resistant strain. These proteins include outer membrane proteins, chaperones, translation factors, and enzymes involved in metabolism (Fernandez-Reyes et al., 2009). Interestingly, some of these isolates incurring biological cost also harbor mutations in the *pmrA/pmrB* TCS (Fernandez-Reyes et al., 2009; Lopez-Rojas et al., 2011; Rolain et al., 2011; Hraiech et al., 2013; Pournaras et al., 2014). Beceiro et al. have recently demonstrated significant biological costs (in virulence and fitness) in colistin-resistant *A. baumannii lpx* mutants (lacking LPSs) compared to colistin-resistant *A. baumannii pmrA/pmrB* mutants (PEtN-modified LPSs) (Beceiro et al., 2014).

### Pseudomonas aeruginosa

The mode of polymyxin resistance in *P. aeruginosa* is very similar to that observed in enteric bacteria. Unlike *A. baumannii*, *P. aeruginosa* has both the *pmrA*/*pmrB* and *phoP*/*phoQ* TCSs, each of which can separately regulate the *arnBCADTEF* operon (McPhee et al., 2003). To date, five TCSs have been described to play a role in polymyxin resistance in *P. aeruginosa* as follows: PmrA/PmrB (McPhee et al., 2003; Moskowitz et al., 2004, 2012; Abraham and Kwon, 2009), PhoP/PhoQ (Macfarlane et al., 1999; Barrow and Kwon, 2009; Schurek et al., 2009; Miller et al., 2011), ParR/ParS (Fernández et al., 2010; Fernandez et al., 2012), ColR/ColS and CprR/CprS (Gutu et al., 2013).

#### PmrA/PmrB and PhoP/PhoQ two-component system-mediated LPS modifications

Several studies have shown that *P. aeruginosa* can develop resistance to polymyxins via the constitutive modification of its LPSs with L-Ara4N, which is stimulated by the *pmrA*/*pmrB* and *phoP*/*phoQ* TCSs (Macfarlane et al., 1999, 2000; McPhee et al., 2003). The constitutive stimulation of these regulatory systems is typically (but not always) induced by mutations (Moskowitz et al., 2004, 2012). The synthesis and transfer of L-Ara4N to the lipid A moiety of LPSs are accomplished by the *arnBCADTEF* operon in *P. aeruginosa*, as has been observed in enteric bacteria.

For the *pmrA*/*pmrB* TCS, several polymyxin-resistant *P. aeruginosa* strains harboring mutations in *pmrB* have been reported (Table 1). These genetic alterations are known to lead to the modification of lipid A with L-Ara4N. Moreover, various resistant isolates harbor double mutations in *pmrB*, and these multimutant strains tend to display high levels of resistance to colistin (Moskowitz et al., 2012). For *P. aeruginosa*, only one study has reported a mutation in *pmrA* that may be responsible for resistance to date (Lee and Ko, 2014), while the rest of the mutations have been mainly localized to PmrB or generally distributed within the cognate regulators of TCSs (Figure 4B; Table 1). Mutations have been observed in different portions of the PmrB domain (Moskowitz et al., 2012; Lee and Ko, 2014), and mutations in the periplasmic and DHP (dimerization and phosphoacceptor) domains, particularly in the histidine box motif, appear to be more significant (Moskowitz et al., 2012), i.e., those close to the putative active histidine residue (H249) tend to evoke greater polymyxin resistance than those distant from this residue (Abraham and Kwon, 2009). In most of the *P. aeruginosa pmrA/pmrB* mutants studied, the MICs of colistin have commonly ranged from 4 to >512 mg/L, mostly performed by the broth microdilution method.

In terms of lipid A modifications in resistant bacteria, there appears to be an absolute relationship of colistin resistance and L-Ara4N addition as well as a significant relationship of this resistance and the loss of secondary lauroyl chain hydroxylation (Miller et al., 2011; Moskowitz et al., 2012). There seems to be wide structural variation in lipid A in polymyxin-resistant *P. aeruginosa* in terms of the presence of either penta- or hexa-acylated lipid A (Moskowitz et al., 2012) in contrast to enteric bacteria, in which a higher degree of acylation is necessary for the addition of L-Ara4N to lipid A (Tran et al., 2005).

Another TCS that has been extensively studied and that also confers resistance in *P. aeruginosa* upon activation is the *phoP*/*phoQ* TCS. Several mutations resulting in in-frame deletions, frameshifts or truncations of the *phoQ* gene that consequently activate the *arnBCADTEF* operon have been reported in resistant strains (Table 1) (Miller et al., 2011; Lee and Ko, 2014). However, not all non-synonymous mutations in *phoQ* result in the constitutive activation of the system (Miller et al., 2011). In contrast to *Salmonella*, polymyxin resistance caused by the activation of *phoP/phoQ* is not dependent on *pmrA/pmrB* in *P. aeruginosa* (Miller et al., 2011). The mutations responsible for PhoP/PhoQ activation appear to occur in *phoQ*, whereas mutations in *phoP* tend to act as primary suppressors in susceptible strains possessing mutations in both genes (Miller et al., 2011). In most of the *P. aeruginosa phoP/phoQ* mutants, colistin MICs usually ranged from 8 to >512 mg/L and were mostly performed by the broth dilution technique. It has been recently reported that mutations in both *pmrB* and *phoQ* result in greater resistant to polymyxins compared to a single mutation in either of the two genes (Owusu-Anim and Kwon, 2012).

Reversions from polymyxin-resistant to polymyxin-susceptible phenotypes among TCS (*pmrA/pmrB* or *phoP/phoQ*) mutants have been observed, and they can occur by the following mechanisms: the reversion of mutant *pmrB* or *phoQ* back to the wild-type gene (loss of mutation), an additional mutation in *phoP* or even the truncation of *phoQ* due to a secondary mutation (Miller et al., 2011; Lee and Ko, 2014). However, there is a high possibility that there are other gene(s) that may act as secondary suppressors when altered because some susceptible strains harbor polymyxin-inducing mutations in *phoQ* with wild-type *phoP* (Miller et al., 2011).

#### Other two-component systems

The ColR/ColS and CprR/CprS TC regulatory systems have been reported to mediate resistance in *P. aeruginosa* either directly or indirectly. Mutations in these two systems have been observed to contribute to high levels of polymyxin resistance in *phoQ* mutant isolates (Gutu et al., 2013). From one viewpoint, the involvement of *colR/colS* and *cprR/cprS* in polymyxin resistance could occur through an interaction with the PhoP/PhoQ regulatory system that enhances *phoQ* activity and influences L-Ara4N modifications, which could lead to high levels of resistance. This interaction could be initiated by the presence of mutations in *colR/colS* and *cprR/cprS* (Table 1). Alternatively, Gutu et al. have suggested that the *colR/colS* and *cprR/cprS* systems may regulate other unknown genes involved in polymyxin resistance in addition to mediating L-Ara4N modifications via mutant *phoQ* (Gutu et al., 2013). This finding indicates that the L-Ara4N modification alone may not be sufficient for the development of polymyxin resistance in *P. aeruginosa*.

Additionally, *cprR/cprS* and another TCS (designated *parR/parS*) have been shown to be involved in adaptive resistance to polymyxins and other antimicrobial peptides. The *cprR/cprS* TCS participates in adaptive resistance by sensing various antimicrobial peptides, such as the synthetic peptide CP28 and polymyxins, and upregulating the *arnBCADTEF* operon (Fernandez et al., 2012). Meanwhile, *parR/parS* can cause the upregulation of the LPS modification operon under sub-inhibitory concentrations of polymyxins and other CAMPs, such as indolicidin but not CP28 (Fernández et al., 2010), demonstrating that the two TCSs are activated by different antimicrobial peptides besides polymyxins. Furthermore, mutations in either ParR or ParS have been observed to cause the constitutive expression of the *arnBCADTEF* operon independent of PmrA/PmrB, resulting in polymyxin resistance. Colistin MIC of 2 mg/L (by agar dilution method) was reported for the clinical strains of *P. aeruginosa* harboring mutations in *parR/parS* genes. This activity has been observed in addition to the mediation of other antibiotic-related genes in *P. aeruginosa* by ParR/ParS (Muller et al., 2011).

#### Other polymyxin resistance determinants in P. aeruginosa

It has been shown that the overexpression of the outer membrane protein OprH in either an Mg^2+^-deficient medium or in a mutant parent strain can result in polymyxin resistance (Young et al., 1992). This is because OprH is a basic protein that binds to divalent cation-binding sites of LPSs, making these sites unavailable for polymyxin binding. However, it has also been suggested that the overexpression of OprH alone is not sufficient for polymyxin resistance in *P. aeruginosa* (Young et al., 1992)

Using transposon mutagenesis, other genes that may play roles in polymyxin resistance have been identified. A total of 17 genes were identified to contribute to intrinsic resistance in *P. aeruginosa* and thus confer supersusceptibility phenotype to polymyxin; the mutant strains showed between 1.5− and 3− fold MIC reduction to polymyxin B compared to the wild-type strain. Notably among these genes, *galU*, *lptC*, *wapR*, and *ssg* participate in LPS biosynthesis-related functions (regulatory functions, metabolism, synthesis and transport) (Fernandez et al., 2013). Disruptions in any of these LPS-mediated genes have been reported to affect the permeability of the outer membrane or to hinder LPS modifications, causing *P. aeruginosa* to be more susceptible to polymyxins (Fernandez et al., 2013).

Other genes that have been recently described to possibly play roles in resistance/susceptibility include those that are able to influence LPS modifications via *pmrA/pmrB* [including a TCS hybrid (PA2583), an arabinose efflux permease-encoding gene (PA5548), a lipoprotein-encoding gene (PA1199), and a hypothetical protein-encoding gene (PA2928)] as well as non-LPS-mediated genes [the response regulator *eraR* (PA1980), the glycosyl transferase *wbpZ* (PA5447) and hypothetical protein-encoding genes (PA4541 and PA1938)]. All of these genes have been mapped against the PAO1 genome (Lee et al., 2014b). The mutant strains display between 0.5- and 4- fold colistin MIC reduction compared to the colistin-resistant wild-type strain with the exception the p*dxB* mutant. The full characterization of these genes would improve the understanding of the complex mechanisms guiding polymyxin resistance in *P. aeruginosa* and help to determine whether these genes are part of the other alternative pathways that have been suggested to mediate colistin resistance in this bacterium (Lee et al., 2014a).

## Intrinsic resistance to polymyxins in bacteria

There are a number of Gram-negative bacteria that are naturally resistant to polymyxins, including but not limited to the following: *Proteus* spp., *Providencia* spp., *Morganella morganii* (all three collectively referred to as the *Proteeae* tribe), *Serratia* spp., *Edwardsiella tarda* and *Burkholderia cepacia* complex (Muyembe et al., 1973; Rozalski et al., 1997; Loutet and Valvano, 2011; Biswas et al., 2012; Samonis et al., 2014). Most of the aforementioned naturally resistant bacteria have LPSs that are modified with L-Ara4N, which may explain their intrinsic resistance (Basu et al., 1986; Boll et al., 1994; Vinogradov et al., 2006).

### Proteus mirabilis

The lipid A and Kdo residue of the LPSs of wild-type *Proteus mirabilis* are known to contain L-Ara4N (Sidorczyk et al., 1983; Boll et al., 1994), which is believed to contribute to the intrinsic resistance of this bacterium to polymyxins. On the other hand, polymyxin-susceptible mutants usually lack L-Ara4N in their LPSs, and those showing reduced resistance contain fewer L-Ara4N substitutions (Kaca et al., 1990; McCoy et al., 2001). Similarly, the genome of *P. mirabilis* has been shown to contain the *eptC* gene, which is involved in the modification of core LPSs with PEtN (Aquilini et al., 2014).

Polymyxin-susceptible mutants generated using transposon mutagenesis have been studied to gain insight into the mechanisms underlying polymyxin resistance in *P. mirabilis*. Some putative loci that may either directly or indirectly affect LPS modifications that have been identified to date include the *sap* operon (encoding a transport protein), the ATPase gene and a putative *O*-acetyltransferase gene (most likely involved in either the biosynthesis or transfer of aminoarabinose) (McCoy et al., 2001). The *galU* gene (involved in L-Ara4N biosynthesis) and a TCS termed *rppA*/*rppB*, which shares close similarity with the *pmrA*/*pmrB* and *phoP*/*phoQ* TCSs and is able to activate the *arnBCADTEF* operon, have been recently discovered to mediate polymyxin resistance in *P. mirabilis* (Wang et al., 2008; Jiang et al., 2010a,b). The inactivation of any of these genes in *P. mirabilis* results in a polymyxin-susceptible phenotype (Wang et al., 2008; Jiang et al., 2010a,b). For example, the transposomic inactivations of P*pmrI* (homologous to *arnA* of *S*. Typhimurium) and *rppA* (homologous to both the *pmrA* and *phoP* of *S*. Typhimurium) genes of *P. mirabilis* resulted in10240- and >160-fold susceptibility to polymyxin B, respectively, compared to the wild-type *P. mirabilis* strain (Wang et al., 2008; Jiang et al., 2010b).

Additionally, genomic analysis of intrinsically colistin-resistant *Morganella morganii*, which is closely related to *Proteus* spp., has revealed that this bacterium possesses an *arnBCADTEF* operon and *eptB* gene known to mediate the modifications of LPSs with L-Ara4N and PEtN, respectively (Olaitan et al., 2014a). The genome of *M. morganii* also contains most of the genes that have been implicated in intrinsic polymyxin resistance in *P. mirabilis*, such as the *rppA*/*rppB* and *phoP/phoQ* TCSs (Olaitan et al., 2014a). It is therefore possible that the *Proteeae* bacteria possess the same underlying mechanisms for polymyxin resistance.

### Serratia marcescens

The inactivation of the *arnB* and *arnC* genes, which are part of the *arnBCADTEF* operon, has been shown to result in polymyxin sensitivity in *Serratia marcescens* mutants (Lin et al., 2014). The *arnB* and *arnC* mutants displayed a reduced susceptibility to polymyxin B with MIC from 2048 to 2 mg/L (a 1024-fold reduction) compared to the wild-type *S. marcescens* strain. This operon has been further reported to be regulated by *phoP*, which is the cognate response regulator of the *phoP/phoQ* TCS (Lin et al., 2014). Thus, these findings show that the modifications of the LPSs of *S. marcescens* as mediated by the *arnBCADTEF* operon are partly or wholly responsible for the intrinsic resistance of this bacterium to polymyxins.

The *arnBCADTEF* operon appears to be constitutively expressed in intrinsically resistant bacteria in contrast to non-intrinsically resistant bacteria. The principal question that remains to be answered is why this is true for these two sets of bacteria. An understanding of this mechanism will greatly aid in the elucidation of the evolution of polymyxin resistance in Gram-negative bacteria.

### Burkholderia species

Generally, members of the genus *Burkholderia* comprising the *Burkholderia cepacia* complex (BCC) and many other *Burkholderia* species are intrinsically resistant to polymyxins (Loutet and Valvano, 2011). L-Ara4N is a predominant part of both the lipid A and Kdo moieties of the LPSs of *Burkholderia* spp., including *B. thailandensis* and *B. pseudomallei* (Isshiki et al., 1998; Silipo et al., 2005; Novem et al., 2009). It is constitutively produced and is essential for these bacteria. A *B. cenocepacia* mutant lacking L-Ara4N has been observed to be highly susceptible to polymyxin B (Ortega et al., 2007). This unique composition together with the contributions of other genes, such as *ispH* (involved in the synthesis of isoprenoids), *rpoE, norM* (an efflux pump-encoding gene), and *hpnJ* (encodes hopanoid), play roles in the multifaceted mechanisms that result in high levels of polymyxin resistance in *Burkholderia cepacia* complex (Loutet and Valvano, 2011; Malott et al., 2012). An excellent review by Loutet et al. on the intrinsic resistance of *Burkholderia cepacia* complex to polymyxins explains the roles of some of these genes (Loutet and Valvano, 2011).

## Conclusions

Resistance to polymyxin antibiotics depends on an intricate mechanism that involves several genes participating in bacterial cell membrane remodeling. However, most of the strategies employed by bacteria target the cell surface, which compromises its integrity or results in its modification, as summarized in Table 2. Although a large amount of work aimed at elucidating the mechanisms underlying polymyxin resistance has been performed, a great deal of information remains to be discovered because resistant strains exist for which the mechanisms underlying their resistance are unknown. Several other candidate genes have also been described, but their contributions to polymyxin resistance have not been fully examined. Such findings are of paramount importance given the current significance of polymyxins in clinical practice and the increased bacterial resistance to these drugs.

**Table 2:** **Strategies employed by Gram-negative bacteria for achieving resistance to polymyxins**.

**Resistance mechanism**	**Genes involved**	**Bacteria**	**References**
Modification of the lipid A or Kdo with aminoarabinose	*arnBCADTEF operon and pmrE*	*Salmonella enterica*, *Klebsiella pneumoniae*, *Escherichia coli*, *Proteeae* bacteria, *Serratia marcescens*, *Pseudomonas aeruginosa* and *Burkholderia cepacia* complex	Vaara et al., 1981; Boll et al., 1994; Nummila et al., 1995; Helander et al., 1996; Rozalski et al., 1997; Trent et al., 2001b; Moskowitz et al., 2004; Yan et al., 2007; Loutet and Valvano, 2011; Lin et al., 2014
Modification of the lipid A with phosphoethanolamine	*pmrC*	*S. enterica*, *K. pneumoniae, E. coli* and *Acinetobacter baumannii*	Zhou et al., 2001; Lee et al., 2004; Kim et al., 2006; Arroyo et al., 2011; Beceiro et al., 2011; Jayol et al., 2014
Activation of LPS-modifying operon by mutations in TCSs	*pmrA/pmrB* and *or phoP/phoQ*	*S. enterica*, *K. pneumoniae, P. aeruginosa* and *A. baumannii*	Roland et al., 1993; Sun et al., 2009; Arroyo et al., 2011; Owusu-Anim and Kwon, 2012; Cannatelli et al., 2014b; Jayol et al., 2014
Inactivation of *phoP/phoQ* negative feedback regulator	*mgrB*	*K. pneumoniae*	Cannatelli et al., 2013; López-Camacho et al., 2013; Gaibani et al., 2014; Olaitan et al., 2014b
Modification of the Kdo with phosphoethanolamine	*eptB*, *phoP/phoQ* and *mgrR*	*E. coli*	Reynolds et al., 2005; Moon and Gottesman, 2009
Increased acylation of lipid A enhancing its modification with aminoarabinose	*lpxM*	*S. enterica*, *K. pneumoniae* and *E. coli*	Tran et al., 2005; Clements et al., 2007; Murray et al., 2007; Velkov et al., 2013b
Trapping of polymyxins by capsule		*K. pneumoniae* and *P. aeruginosa*	Campos et al., 2004; Llobet et al., 2008
Efflux pump	*acrAB* and *kpnEF*	*K. pneumoniae*	Padilla et al., 2010; Srinivasan and Rajamohan, 2013
Loss of LPS	*lpxA*, *lpxC* and *lpxD*	*A. baumannii*	Moffatt et al., 2010, 2011
Glycosylation of lipid A with hexosamine		*A. baumannii*	Pelletier et al., 2013
Acquired/adaptive resistance to polymyxins through LPS modification with aminoarabinose	*colR/colS*, *cprR/cprS* and *parR/parS*	*P. aeruginosa*	Fernández et al., 2010; Muller et al., 2011; Fernandez et al., 2012; Gutu et al., 2013
Overexpression of outer membrane protein OprH	*oprH*	*P. aeruginosa*	Young et al., 1992

## Author contributions

Conceived and designed the study: Jean-Marc Rolain and Serge Morand; analyzed the data: Abiola O. Olaitan and Jean-Marc Rolain; wrote the paper: Abiola O. Olaitan and Jean-Marc Rolain.

### Conflict of interest statement

The authors declare that the research was conducted in the absence of any commercial or financial relationships that could be construed as a potential conflict of interest.

## References

[B1] Abraham N. Kwon D. H. (2009). A single amino acid substitution in PmrB is associated with polymyxin B resistance in clinical isolate of *Pseudomonas aeruginosa*. FEMS Microbiol. Lett. 298, 249–254. 10.1111/j.1574-6968.2009.01720.x 19663916

[B2] Adams M. D. Nickel G. C. Bajaksouzian S. Lavender H. Murthy A. R. Jacobs M. R.. (2009). Resistance to colistin in *Acinetobacter baumannii* associated with mutations in the PmrAB two-component system. Antimicrob. Agents Chemother. 53, 3628–3634. 10.1128/AAC.00284-09 19528270 PMC2737849

[B3] Agerso Y. Torpdahl M. Zachariasen C. Seyfarth A. Hammerum A. M. Nielsen E. M. (2012). Tentative colistin epidemiological cut-off value for *Salmonella* spp. Foodborne Pathog. Dis. 9, 367–369. 10.1089/fpd.2011.1015 22300222

[B4] Aguirre A. Lejona S. Vescovi E. G. Soncini F. C. (2000). Phosphorylated PmrA interacts with the promoter region of ugd in *Salmonella enterica* serovar typhimurium. J. Bacteriol. 182, 3874–3876. 10.1128/JB.182.13.3874-3876.2000 10851011 PMC94567

[B5] Aquilini E. Merino S. Knirel Y. A. Regue M. Tomas J. M. (2014). Functional identification of *Proteus mirabilis* eptC gene encoding a core lipopolysaccharide phosphoethanolamine transferase. Int. J. Mol. Sci. 15, 6689–6702. 10.3390/ijms15046689 24756091 PMC4013655

[B6] Arduino S. M. Quiroga M. P. Ramirez M. S. Merkier A. K. Errecalde L. Di M. A.. (2012). Transposons and integrons in colistin-resistant clones of *Klebsiella pneumoniae* and *Acinetobacter baumannii* with epidemic or sporadic behaviour. J. Med. Microbiol. 61, 1417–1420. 10.1099/jmm.0.038968-0 22723256

[B7] Arroyo L. A. Herrera C. M. Fernandez L. Hankins J. V. Trent M. S. Hancock R. E. (2011). The pmrCAB operon mediates polymyxin resistance in *Acinetobacter baumannii* ATCC 17978 and clinical isolates through phosphoethanolamine modification of lipid A. Antimicrob. Agents Chemother. 55, 3743–3751. 10.1128/AAC.00256-11 21646482 PMC3147623

[B8] Barchiesi J. Espariz M. Checa S. K. Soncini F. C. (2009). Downregulation of RpoN-controlled genes protects *Salmonella* cells from killing by the cationic antimicrobial peptide polymyxin B. FEMS Microbiol. Lett. 291, 73–79. 10.1111/j.1574-6968.2008.01437.x 19076233

[B9] Barin J. Martins A. F. Heineck B. L. Barth A. L. Zavascki A. P. (2013). Hetero- and adaptive resistance to polymyxin B in OXA-23-producing carbapenem-resistant *Acinetobacter baumannii* isolates. Ann. Clin. Microbiol. Antimicrob. 12, 15. 10.1186/1476-0711-12-15 23819554 PMC3707782

[B10] Barrow K. Kwon D. H. (2009). Alterations in two-component regulatory systems of phoPQ and pmrAB are associated with polymyxin B resistance in clinical isolates of *Pseudomonas aeruginosa*. Antimicrob. Agents Chemother. 53, 5150–5154. 10.1128/AAC.00893-09 19752280 PMC2786363

[B11] Basu S. Radziejewska-Lebrecht J. Mayer H. (1986). Lipopolysaccharide of Providencia rettgeri. Chemical studies and taxonomical implications. Arch. Microbiol. 144, 213–218. 10.1007/BF00410949 3524498

[B12] Beceiro A. Llobet E. Aranda J. Bengoechea J. A. Doumith M. Hornsey M.. (2011). Phosphoethanolamine modification of lipid A in colistin-resistant variants of *Acinetobacter baumannii* mediated by the pmrAB two-component regulatory system. Antimicrob. Agents Chemother. 55, 3370–3379. 10.1128/AAC.00079-11 21576434 PMC3122444

[B13] Beceiro A. Moreno A. Fernandez N. Vallejo J. A. Aranda J. Adler B.. (2014). Biological cost of different mechanisms of colistin resistance and their impact on virulence in *Acinetobacter baumannii*. Antimicrob. Agents Chemother. 58, 518–526. 10.1128/AAC.01597-13 24189257 PMC3910726

[B14] Biswas S. Brunel J. M. Dubus J. C. Reynaud-Gaubert M. Rolain J. M. (2012). Colistin: an update on the antibiotic of the 21st century. Expert Rev. Anti. Infect. Ther. 10, 917–934. 10.1586/eri.12.78 23030331

[B15] Boll M. Radziejewska-Lebrecht J. Warth C. Krajewska-Pietrasik D. Mayer H. (1994). 4-Amino-4-deoxy-L-arabinose in LPS of enterobacterial R-mutants and its possible role for their polymyxin reactivity. FEMS Immunol. Med. Microbiol. 8, 329–341. 8061656 10.1111/j.1574-695X.1994.tb00460.x

[B16] Cai Y. Chai D. Wang R. Liang B. Bai N. (2012). Colistin resistance of *Acinetobacter baumannii*: clinical reports, mechanisms and antimicrobial strategies. J. Antimicrob. Chemother. 67, 1607–1615. 10.1093/jac/dks084 22441575

[B17] Campos M. A. Vargas M. A. Regueiro V. Llompart C. M. Alberti S. Bengoechea J. A. (2004). Capsule polysaccharide mediates bacterial resistance to antimicrobial peptides. Infect. Immun. 72, 7107–7114. 10.1128/IAI.72.12.7107-7114.2004 15557634 PMC529140

[B18] Cannatelli A. D'Andrea M. M. Giani T. Di P. V. Arena F. Ambretti S.. (2013). *In vivo* emergence of colistin resistance in *Klebsiella pneumoniae* producing KPC-type carbapenemases mediated by insertional inactivation of the PhoQ/PhoP mgrB regulator. Antimicrob. Agents Chemother. 57, 5521–5526. 10.1128/AAC.01480-13 23979739 PMC3811314

[B19] Cannatelli A. Giani T. D'Andrea M. M. Di Pilato V. Arena F. Conte V.. (2014a). MgrB inactivation is a common mechanism of colistin resistance in KPC carbapenemase-producing *Klebsiella pneumoniae* of clinical origin. Antimicrob. Agents Chemother. 58, 5696–5703. 10.1128/AAC.03110-14 25022583 PMC4187966

[B20] Cannatelli A. Di Pilato V. Giani T. Arena F. Ambretti S. Gaibani P.. (2014b). *In vivo* evolution to colistin resistance by PmrB sensor kinase mutation in KPC carbapenemase-producing *Klebsiella pneumoniae* associated with low-dosage colistin treatment. Antimicrob. Agents Chemother. 58, 4399–4403. 10.1128/AAC.02555-14 24841267 PMC4136067

[B21] Cheng H. Y. Chen Y. F. Peng H. L. (2010). Molecular characterization of the PhoPQ-PmrD-PmrAB mediated pathway regulating polymyxin B resistance in *Klebsiella pneumoniae* CG43. J. Biomed. Sci. 17:60. 10.1186/1423-0127-17-60 20653976 PMC2919465

[B22] Choi M. J. Ko K. S. (2014). Mutant prevention concentrations of colistin for *Acinetobacter baumannii*, *Pseudomonas aeruginosa* and *Klebsiella pneumoniae* clinical isolates. J. Antimicrob. Chemother. 69, 275–277. 10.1093/jac/dkt315 23997018

[B23] Clements A. Tull D. Jenney A. W. Farn J. L. Kim S. H. Bishop R. E.. (2007). Secondary acylation of *Klebsiella pneumoniae* lipopolysaccharide contributes to sensitivity to antibacterial peptides. J. Biol. Chem. 282, 15569–15577. 10.1074/jbc.M701454200 17371870 PMC5007121

[B24] Coornaert A. Lu A. Mandin P. Springer M. Gottesman S. Guillier M. (2010). MicA sRNA links the PhoP regulon to cell envelope stress. Mol. Microbiol. 76, 467–479. 10.1111/j.1365-2958.2010.07115.x 20345657 PMC2925231

[B25] de J. A. Thomas V. Simjee S. Godinho K. Schiessl B. Klein U.. (2012). Pan-European monitoring of susceptibility to human-use antimicrobial agents in enteric bacteria isolated from healthy food-producing animals. J. Antimicrob. Chemother. 67, 638–651. 10.1093/jac/dkr539 22210758

[B26] Diene S. M. Merhej V. Henry M. El F. A. Roux V. Robert C.. (2013). The rhizome of the multidrug-resistant *Enterobacter* aerogenes genome reveals how new “killer bugs” are created because of a sympatric lifestyle. Mol. Biol. Evol. 30, 369–383. 10.1093/molbev/mss236 23071100

[B27] EMEA. (2002). Colistin: Summary report (1), EMEA/MRL/016/95-FINAL. London: The European Agency for the Evaluation of Medicinal Products, Committee for Veterinary Medicinal Products.

[B146] Ernst R. K. Guina T. Miller S. I. (2001). Salmonella Typhimurium outer membrane remodeling: role in resistance to host innate immunity. Microbes Infect. 3, 1327–1334. 11755422 10.1016/s1286-4579(01)01494-0

[B28] Falagas M. E. Michalopoulos A. (2006). Polymyxins: old antibiotics are back. Lancet 367, 633–634. 10.1016/S0140-6736(06)68241-X 16503447

[B29] Falagas M. E. Rafailidis P. I. Matthaiou D. K. (2010). Resistance to polymyxins: Mechanisms, frequency and treatment options. Drug Resist. Updat. 13, 132–138. 10.1016/j.drup.2010.05.002 20843473

[B30] Fernandez L. Alvarez-Ortega C. Wiegand I. Olivares J. Kocincova D. Lam J. S.. (2013). Characterization of the polymyxin B resistome of *Pseudomonas aeruginosa*. Antimicrob. Agents Chemother. 57, 110–119. 10.1128/AAC.01583-12 23070157 PMC3535977

[B149] Fernández L. Gooderham W. J. Bains M. McPhee J. B. Wiegand I. Hancock R. E. W. (2010). Adaptive resistance to the “last hope” antibiotics polymyxin B and colistin in Pseudomonas aeruginosa is mediated by the novel two-component regulatory system ParR-ParS. Antimicrob. Agents Chemother. 54, 3372–3382. 10.1128/AAC.00242-10 20547815 PMC2916309

[B31] Fernandez L. Jenssen H. Bains M. Wiegand I. Gooderham W. J. Hancock R. E. (2012). The two-component system CprRS senses cationic peptides and triggers adaptive resistance in *Pseudomonas aeruginosa* independently of ParRS. Antimicrob. Agents Chemother. 56, 6212–6222. 10.1128/AAC.01530-12 23006746 PMC3497197

[B32] Fernandez-Reyes M. Rodriguez-Falcon M. Chiva C. Pachon J. Andreu D. Rivas L. (2009). The cost of resistance to colistin in *Acinetobacter baumannii*: a proteomic perspective. Proteomics 9, 1632–1645. 10.1002/pmic.200800434 19253303

[B33] Gaibani P. Lombardo D. Lewis R. E. Mercuri M. Bonora S. Landini M. P.. (2014). *In vitro* activity and post-antibiotic effects of colistin in combination with other antimicrobials against colistin-resistant KPC-producing *Klebsiella pneumoniae* bloodstream isolates. J. Antimicrob. Chemother. 69, 1856–1865. 10.1093/jac/dku065 24648503

[B34] Guckes K. R. Kostakioti M. Breland E. J. Gu A. P. Shaffer C. L. Martinez C. R. III. (2013). Strong cross-system interactions drive the activation of the QseB response regulator in the absence of its cognate sensor. Proc. Natl. Acad. Sci. U.S.A. 110, 16592–16597. 10.1073/pnas.1315320110 24062463 PMC3799328

[B35] Gunn J. S. (2001). Bacterial modification of LPS and resistance to antimicrobial peptides. J. Endotoxin. Res. 7, 57–62. 10.1177/09680519010070011001 11521084

[B36] Gunn J. S. Lim K. B. Krueger J. Kim K. Guo L. Hackett M.. (1998). PmrA-PmrB-regulated genes necessary for 4-aminoarabinose lipid A modification and polymyxin resistance. Mol. Microbiol. 27, 1171–1182. 10.1046/j.1365-2958.1998.00757.x 9570402

[B37] Gunn J. S. Miller S. I. (1996). PhoP-PhoQ activates transcription of pmrAB, encoding a two-component regulatory system involved in *Salmonella* typhimurium antimicrobial peptide resistance. J. Bacteriol. 178, 6857–6864. 8955307 10.1128/jb.178.23.6857-6864.1996 PMC178586

[B38] Gunn J. S. Ryan S. S. Van Velkinburgh J. C. Ernst R. K. Miller S. I. (2000). Genetic and functional analysis of a PmrA-PmrB-regulated locus necessary for lipopolysaccharide modification, antimicrobial peptide resistance, and oral virulence of *Salmonella enterica* serovar typhimurium. Infect. Immun. 68, 6139–6146. 10.1128/IAI.68.11.6139-6146.2000 11035717 PMC97691

[B39] Guo L. Lim K. B. Gunn J. S. Bainbridge B. Darveau R. P. Hackett M.. (1997). Regulation of lipid A modifications by *Salmonella* typhimurium virulence genes phoP-phoQ. Science 276, 250–253. 10.1126/science.276.5310.250 9092473

[B40] Gutu A. D. Sgambati N. Strasbourger P. Brannon M. K. Jacobs M. A. Haugen E.. (2013). Polymyxin resistance of *Pseudomonas aeruginosa* phoQ mutants is dependent on additional two-component regulatory systems. Antimicrob. Agents Chemother. 57, 2204–2215. 10.1128/AAC.02353-12 23459479 PMC3632916

[B41] Hayakawa K. Marchaim D. Divine G. W. Pogue J. M. Kumar S. Lephart P.. (2012). Growing prevalence of Providencia stuartii associated with the increased usage of colistin at a tertiary health care center. Int. J. Infect. Dis. 16, e646–e648. 10.1016/j.ijid.2012.05.1029 22818111

[B42] Helander I. M. Kato Y. Kilpelainen I. Kostiainen R. Lindner B. Nummila K.. (1996). Characterization of lipopolysaccharides of polymyxin-resistant and polymyxin-sensitive *Klebsiella pneumoniae* O3. Eur. J. Biochem. 237, 272–278. 10.1111/j.1432-1033.1996.0272n.x 8620884

[B43] Helander I. M. Kilpelainen I. Vaara M. (1994). Increased substitution of phosphate groups in lipopolysaccharides and lipid A of the polymyxin-resistant pmrA mutants of *Salmonella* typhimurium: a 31P-NMR study. Mol. Microbiol. 11, 481–487. 10.1111/j.1365-2958.1994.tb00329.x 8152372

[B44] Herrera C. M. Hankins J. V. Trent M. S. (2010). Activation of PmrA inhibits LpxT-dependent phosphorylation of lipid A promoting resistance to antimicrobial peptides. Mol. Microbiol. 76, 1444–1460. 10.1111/j.1365-2958.2010.07150.x 20384697 PMC2904496

[B45] Hood M. I. Becker K. W. Roux C. M. Dunman P. M. Skaar E. P. (2013). genetic determinants of intrinsic colistin tolerance in *Acinetobacter baumannii*. Infect. Immun. 81, 542–551. 10.1128/IAI.00704-12 23230287 PMC3553813

[B46] Hraiech S. Roch A. Lepidi H. Atieh T. Audoly G. Rolain J. M.. (2013). Impaired virulence and fitness of a colistin-resistant clinical isolate of *Acinetobacter baumannii* in a rat model of pneumonia. Antimicrob. Agents Chemother. 57, 5120–5121. 10.1128/AAC.00700-13 23836181 PMC3811450

[B47] Isshiki Y. Kawahara K. Zahringer U. (1998). Isolation and characterisation of disodium (4-amino-4-deoxy-beta-L- arabinopyranosyl)-(1–>8)-(D-glycero-alpha-D-talo-oct-2-ulopyranosylona te)- (2–>4)-(methyl 3-deoxy-D-manno-oct-2-ulopyranosid)onate from the lipopolysaccharide of *Burkholderia cepacia*. Carbohydr. Res. 313, 21–27. 10.1016/S0008-6215(98)00179-7 9861699

[B48] Jayol A. Poirel L. Brink A. Villegas M.-V. Yilmaz M. Nordmann P. (2014). Resistance to colistin associated to a single amino acid change in protein PmrB among *Klebsiella pneumoniae* isolates of worldwide origin. Antimicrob. Agents Chemother. 58, 4762–4766. 10.1128/AAC.00084-14 24914122 PMC4136042

[B49] Jiang S. S. Lin T. Y. Wang W. B. Liu M. C. Hsueh P. R. Liaw S. J. (2010a). Characterization of UDP-glucose dehydrogenase and UDP-glucose pyrophosphorylase mutants of *Proteus mirabilis*: defectiveness in polymyxin B resistance, swarming, and virulence. Antimicrob. Agents Chemother. 54, 2000–2009. 10.1128/AAC.01384-09 20160049 PMC2863647

[B50] Jiang S. S. Liu M. C. Teng L. J. Wang W. B. Hsueh P. R. Liaw S. J. (2010b). *Proteus mirabilis* pmrI, an RppA-regulated gene necessary for polymyxin B resistance, biofilm formation, and urothelial cell invasion. Antimicrob. Agents Chemother. 54, 1564–1571. 10.1128/AAC.01219-09 20123999 PMC2849355

[B51] Johansen H. K. Moskowitz S. M. Ciofu O. Pressler T. Hoiby N. (2008). Spread of colistin resistant non-mucoid *Pseudomonas aeruginosa* among chronically infected Danish cystic fibrosis patients. J. Cyst. Fibros. 7, 391–397. 10.1016/j.jcf.2008.02.003 18358794

[B52] Jones J. W. Shaffer S. A. Ernst R. K. Goodlett D. R. Turecek F. (2008). Determination of pyrophosphorylated forms of lipid A in Gram-negative bacteria using a multivaried mass spectrometric approach. Proc. Natl. Acad. Sci. U.S.A. 105, 12742–12747. 10.1073/pnas.0800445105 18753624 PMC2529124

[B53] Kaca W. Radziejewska-Lebrecht J. Bhat U. R. (1990). Effect of polymyxins on the lipopolysaccharide-defective mutants of *Proteus mirabilis*. Microbios 61, 23–32. 2156134

[B54] Kato A. Chen H. D. Latifi T. Groisman E. A. (2012). Reciprocal control between a bacterium's regulatory system and the modification status of its lipopolysaccharide. Mol. Cell 47, 897–908. 10.1016/j.molcel.2012.07.017 22921935 PMC3465083

[B55] Kato A. Latifi T. Groisman E. A. (2003). Closing the loop: the PmrA/PmrB two-component system negatively controls expression of its posttranscriptional activator PmrD. Proc. Natl. Acad. Sci. U.S.A. 100, 4706–4711. 10.1073/pnas.0836837100 12676988 PMC153620

[B56] Kawasaki K. China K. Nishijima M. (2007). Release of the lipopolysaccharide deacylase PagL from latency compensates for a lack of lipopolysaccharide aminoarabinose modification-dependent resistance to the antimicrobial peptide polymyxin B in *Salmonella enterica*. J. Bacteriol. 189, 4911–4919. 10.1128/JB.00451-07 17483225 PMC1913436

[B147] Kawasaki K. Ernst R. K. Miller S. I. (2005). Inhibition of Salmonella enterica serovar Typhimurium lipopolysaccharide deacylation by aminoarabinose membrane modification. J. Bacteriol. 187, 2448–2457. 10.1128/JB.187.7.2448-2457.2005 15774888 PMC1065228

[B57] Kazmierczak M. J. Wiedmann M. Boor K. J. (2005). Alternative sigma factors and their roles in bacterial virulence. Microbiol. Mol. Biol. Rev. 69, 527–543. 10.1128/MMBR.69.4.527-543.2005 16339734 PMC1306804

[B58] Kempf I. Fleury M. A. Drider D. Bruneau M. Sanders P. Chauvin C.. (2013). What do we know about resistance to colistin in *Enterobacteriaceae* in avian and pig production in Europe? Int. J. Antimicrob. Agents 42, 379–383. 10.1016/j.ijantimicag.2013.06.012 24076115

[B59] Kim S. H. Jia W. Parreira V. R. Bishop R. E. Gyles C. L. (2006). Phosphoethanolamine substitution in the lipid A of *Escherichia coli* O157: H7 and its association with PmrC. Microbiology 152, 657–666. 10.1099/mic.0.28692-0 16514146

[B60] Kim S. Y. Choi H. J. Ko K. S. (2014a). Differential expression of two-component systems, pmrAB and phoPQ, with different growth phases of *Klebsiella pneumoniae* in the presence or absence of colistin. Curr. Microbiol. 69, 37–41. 10.1007/s00284-014-0549-0 24577612

[B61] Kim Y. Bae I. K. Lee H. Jeong S. H. Yong D. Lee K. (2014b). *In vivo* emergence of colistin resistance in *Acinetobacter baumannii* clinical isolates of sequence type 357 during colistin treatment. Diagn. Microbiol. Infect. Dis. 79, 362–366. 10.1016/j.diagmicrobio.2014.03.027 24809861

[B62] Kox L. F. Wosten M. M. Groisman E. A. (2000). A small protein that mediates the activation of a two-component system by another two-component system. EMBO J. 19, 1861–1872. 10.1093/emboj/19.8.1861 10775270 PMC302009

[B63] Lacour S. Bechet E. Cozzone A. J. Mijakovic I. Grangeasse C. (2008). Tyrosine phosphorylation of the UDP-glucose dehydrogenase of *Escherichia coli* is at the crossroads of colanic acid synthesis and polymyxin resistance. PLoS ONE 3:e3053. 10.1371/journal.pone.0003053 18725960 PMC2516531

[B64] Lacour S. Doublet P. Obadia B. Cozzone A. J. Grangeasse C. (2006). A novel role for protein-tyrosine kinase Etk from *Escherichia coli* K-12 related to polymyxin resistance. Res. Microbiol. 157, 637–641. 10.1016/j.resmic.2006.01.003 16814990

[B65] Lean S. S. Suhaili Z. Ismail S. Rahman N. I. Othman N. Abdullah F. H.. (2014). Prevalence and genetic characterization of carbapenem- and polymyxin-resistant *Acinetobacter baumannii* Isolated from a tertiary hospital in Terengganu, Malaysia. ISRN. Microbiol. 2014, 953417. 10.1155/2014/953417 25006521 PMC3977555

[B66] Lee H. Hsu F. F. Turk J. Groisman E. A. (2004). The PmrA-regulated pmrC gene mediates phosphoethanolamine modification of lipid A and polymyxin resistance in *Salmonella enterica*. J. Bacteriol. 186, 4124–4133. 10.1128/JB.186.13.4124-4133.2004 15205413 PMC421605

[B67] Lee J.-Y. Chung E. S. Na I. Y. Kim H. Shin D. Ko K. S. (2014a). Development of colistin resistance in pmrA-, phoP-, parR- and cprR-inactivated mutants of *Pseudomonas aeruginosa*. J. Antimicrob. Chemother. 69, 2966–2971. 10.1093/jac/dku238 24994873

[B68] Lee J. Y. Ko K. S. (2014). Mutations and expression of PmrAB and PhoPQ related with colistin resistance in *Pseudomonas aeruginosa* clinical isolates. Diagn. Microbiol. Infect. Dis. 78, 271–276. 10.1016/j.diagmicrobio.2013.11.027 24412662

[B69] Lee J. Y. Na I. Y. Park Y. K. Ko K. S. (2014b). Genomic variations between colistin-susceptible and -resistant *Pseudomonas aeruginosa* clinical isolates and their effects on colistin resistance. J. Antimicrob. Chemother. 69, 1248–1256. 10.1093/jac/dkt531 24474431

[B70] Lesho E. Yoon E. J. McGann P. Snesrud E. Kwak Y. Milillo M.. (2013). Emergence of colistin-resistance in extremely drug-resistant *Acinetobacter baumannii* containing a novel pmrCAB operon during colistin therapy of wound infections. J. Infect. Dis. 208, 1142–1151. 10.1093/infdis/jit293 23812239

[B71] Li J. Rayner C. R. Nation R. L. Owen R. J. Spelman D. Tan K. E.. (2006). Heteroresistance to colistin in multidrug-resistant *Acinetobacter baumannii*. Antimicrob. Agents Chemother. 50, 2946–2950. 10.1128/AAC.00103-06 16940086 PMC1563544

[B72] Lim L. M. Ly N. Anderson D. Yang J. C. Macander L. Jarkowski A. III. (2010). Resurgence of colistin: a review of resistance, toxicity, pharmacodynamics, and dosing. Pharmacotherapy 30, 1279–1291. 10.1592/phco.30.12.1279 21114395 PMC4410713

[B73] Lin Q. Y. Tsai Y.-L. Liu M.-C. Lin W.-C. Hsueh P.-R. Liaw S.-J. (2014). *Serratia marcescens* arn, a PhoP-regulated locus necessary for polymyxin B resistance. Antimicrob. Agents Chemother. 58, 5181–5190. 10.1128/AAC.00013-14 24957827 PMC4135846

[B74] Lippa A. M. Goulian M. (2009). Feedback inhibition in the PhoQ/PhoP signaling system by a membrane peptide. PLoS. Genet. 5:e1000788. 10.1371/journal.pgen.1000788 20041203 PMC2789325

[B75] Lippa A. M. Goulian M. (2012). Perturbation of the oxidizing environment of the periplasm stimulates the PhoQ/PhoP system in *Escherichia coli*. J. Bacteriol. 194, 1457–1463. 10.1128/JB.06055-11 22267510 PMC3294871

[B76] Llobet E. Campos M. A. Gimenez P. Moranta D. Bengoechea J. A. (2011). Analysis of the networks controlling the antimicrobial-peptide-dependent induction of *Klebsiella pneumoniae* virulence factors. Infect. Immun. 79, 3718–3732. 10.1128/IAI.05226-11 21708987 PMC3165464

[B77] Llobet E. Tomas J. M. Bengoechea J. A. (2008). Capsule polysaccharide is a bacterial decoy for antimicrobial peptides. Microbiology 154, 3877–3886. 10.1099/mic.0.2008/022301-0 19047754

[B78] López-Camacho E. Gómez-Gil R. Tobes R. Manrique M. Lorenzo M. Galván B.. (2013). Genomic analysis of the emergence and evolution of multidrug resistance during a *Klebsiella pneumoniae* outbreak including carbapenem and colistin resistance. J. Antimicrob. Chemother. 69, 632–636. 10.1093/jac/dkt419 24155060

[B79] Lopez-Rojas R. Dominguez-Herrera J. McConnell M. J. Docobo-Perez F. Smani Y. Fernandez-Reyes M.. (2011). Impaired virulence and *in vivo* fitness of colistin-resistant *Acinetobacter baumannii*. J. Infect. Dis. 203, 545–548. 10.1093/infdis/jiq086 21216865 PMC3071218

[B80] Loutet S. A. Valvano M. A. (2011). Extreme antimicrobial peptide and polymyxin B resistance in the genus *Burkholderia*. Front. Cell Infect. Microbiol. 1:6. 10.3389/fcimb.2011.00006 22919572 PMC3417367

[B81] Macfarlane E. L. Kwasnicka A. Hancock R. E. (2000). Role of *Pseudomonas aeruginosa* PhoP-phoQ in resistance to antimicrobial cationic peptides and aminoglycosides. Microbiology 146(Pt 10), 2543–2554. 11021929 10.1099/00221287-146-10-2543

[B82] Macfarlane E. L. Kwasnicka A. Ochs M. M. Hancock R. E. (1999). PhoP-PhoQ homologues in *Pseudomonas aeruginosa* regulate expression of the outer-membrane protein OprH and polymyxin B resistance. Mol. Microbiol. 34, 305–316. 10.1046/j.1365-2958.1999.01600.x 10564474

[B83] Malott R. J. Steen-Kinnaird B. R. Lee T. D. Speert D. P. (2012). Identification of hopanoid biosynthesis genes involved in polymyxin resistance in *Burkholderia* multivorans. Antimicrob. Agents Chemother. 56, 464–471. 10.1128/AAC.00602-11 22006009 PMC3256039

[B84] Mammina C. Bonura C. Di B. F. Aleo A. Fasciana T. Sodano C.. (2012). Ongoing spread of colistin-resistant *Klebsiella pneumoniae* in different wards of an acute general hospital, Italy, June to December 2011. Euro. Surveill 17. 22913977

[B85] McCoy A. J. Liu H. Falla T. J. Gunn J. S. (2001). Identification of *Proteus mirabilis* mutants with increased sensitivity to antimicrobial peptides. Antimicrob. Agents Chemother. 45, 2030–2037. 10.1128/AAC.45.7.2030-2037.2001 11408219 PMC90596

[B86] McPhee J. B. Lewenza S. Hancock R. E. (2003). Cationic antimicrobial peptides activate a two-component regulatory system, PmrA-PmrB, that regulates resistance to polymyxin B and cationic antimicrobial peptides in *Pseudomonas aeruginosa*. Mol. Microbiol. 50, 205–217. 10.1046/j.1365-2958.2003.03673.x 14507375

[B87] Merkier A. K. Rodriguez M. C. Togneri A. Brengi S. Osuna C. Pichel M.. (2013). Outbreak of a cluster with epidemic behavior due to *Serratia marcescens* after colistin administration in a hospital setting. J. Clin. Microbiol. 51, 2295–2302. 10.1128/JCM.03280-12 23698525 PMC3697717

[B88] Miller A. K. Brannon M. K. Stevens L. Johansen H. K. Selgrade S. E. Miller S. I.. (2011). PhoQ mutations promote lipid A modification and polymyxin resistance of *Pseudomonas aeruginosa* found in colistin-treated cystic fibrosis patients. Antimicrob. Agents Chemother. 55, 5761–5769. 10.1128/AAC.05391-11 21968359 PMC3232818

[B89] Mitrophanov A. Y. Jewett M. W. Hadley T. J. Groisman E. A. (2008). Evolution and dynamics of regulatory architectures controlling polymyxin B resistance in enteric bacteria. PLoS. Genet. 4:e1000233. 10.1371/journal.pgen.1000233 18949034 PMC2565834

[B90] Moffatt J. H. Harper M. Adler B. Nation R. L. Li J. Boyce J. D. (2011). Insertion sequence ISAba11 is involved in colistin resistance and loss of lipopolysaccharide in *Acinetobacter baumannii*. Antimicrob. Agents Chemother. 55, 3022–3024. 10.1128/AAC.01732-10 21402838 PMC3101452

[B91] Moffatt J. H. Harper M. Harrison P. Hale J. D. Vinogradov E. Seemann T.. (2010). Colistin resistance in *Acinetobacter baumannii* is mediated by complete loss of lipopolysaccharide production. Antimicrob. Agents Chemother. 54, 4971–4977. 10.1128/AAC.00834-10 20855724 PMC2981238

[B92] Moon K. Gottesman S. (2009). A PhoQ/P-regulated small RNA regulates sensitivity of *Escherichia coli* to antimicrobial peptides. Mol. Microbiol. 74, 1314–1330. 10.1111/j.1365-2958.2009.06944.x 19889087 PMC2841474

[B93] Morales A. S. Fragoso de A. J. de Moura Gomes V. T. Reis Costa A. T. dos Prazeres R. D. Porfida Ferreira T. S.. (2012). Colistin resistance in *Escherichia coli* and *Salmonella enterica* strains isolated from swine in Brazil. ScientificWorldJournal 2012:109795. 10.1100/2012/109795 22973166 PMC3432351

[B94] Moskowitz S. M. Brannon M. K. Dasgupta N. Pier M. Sgambati N. Miller A. K.. (2012). PmrB mutations promote polymyxin resistance of *Pseudomonas aeruginosa* isolated from colistin-treated cystic fibrosis patients. Antimicrob. Agents Chemother. 56, 1019–1030. 10.1128/AAC.05829-11 22106224 PMC3264203

[B95] Moskowitz S. M. Ernst R. K. Miller S. I. (2004). PmrAB, a two-component regulatory system of *Pseudomonas aeruginosa* that modulates resistance to cationic antimicrobial peptides and addition of aminoarabinose to lipid A. J. Bacteriol. 186, 575–579. 10.1128/JB.186.2.575-579.2004 14702327 PMC305751

[B96] Muller C. Plesiat P. Jeannot K. (2011). A two-component regulatory system interconnects resistance to polymyxins, aminoglycosides, fluoroquinolones, and beta-lactams in *Pseudomonas aeruginosa*. Antimicrob. Agents Chemother. 55, 1211–1221. 10.1128/AAC.01252-10 21149619 PMC3067119

[B97] Murray S. R. Ernst R. K. Bermudes D. Miller S. I. Low K. B. (2007). pmrA(Con) confers pmrHFIJKL-dependent EGTA and polymyxin resistance on msbB *Salmonella* by decorating lipid A with phosphoethanolamine. J. Bacteriol. 189, 5161–5169. 10.1128/JB.01969-06 17449614 PMC1951887

[B98] Muyembe T. Vandepitte J. Desmyter J. (1973). Natural colistin resistance in Edwardsiella tarda. Antimicrob. Agents Chemother. 4, 521–524. 10.1128/AAC.4.5.521 4791485 PMC444588

[B99] Nation R. L. Li J. (2009). Colistin in the 21st century. Curr. Opin. Infect. Dis. 22, 535–543. 10.1097/QCO.0b013e328332e672 19797945 PMC2869076

[B100] Nikaido H. (2003). Molecular basis of bacterial outer membrane permeability revisited. Microbiol. Mol. Biol. Rev. 67, 593–656. 10.1128/MMBR.67.4.593-656.2003 14665678 PMC309051

[B101] Novem V. Shui G. Wang D. Bendt A. K. Sim S. H. Liu Y.. (2009). Structural and biological diversity of lipopolysaccharides from *Burkholderia pseudomallei* and *Burkholderia thailandensis*. Clin. Vaccine Immunol. 16, 1420–1428. 10.1128/CVI.00472-08 19692625 PMC2756838

[B102] Nummila K. Kilpelainen I. Zahringer U. Vaara M. Helander I. M. (1995). Lipopolysaccharides of polymyxin B-resistant mutants of *Escherichia coli* are extensively substituted by 2-aminoethyl pyrophosphate and contain aminoarabinose in lipid A. Mol. Microbiol. 16, 271–278. 10.1111/j.1365-2958.1995.tb02299.x 7565089

[B103] Olaitan A. O. Diene S. M. Gupta S. K. Adler A. Assous M. V. Rolain J.-M. (2014a). Genome analysis of NDM-1 producing *Morganella morganii* clinical isolate. Expert Rev. Anti. Infect. Ther. 12, 1297–1305. 10.1586/14787210.2014.944504 25081858

[B104] Olaitan A. O. Diene S. M. Kempf M. Berrazeg M. Bakour S. Gupta S. K.. (2014b). Worldwide emergence of colistin resistance in *Klebsiella pneumoniae* from healthy humans and patients in Lao PDR, Thailand, Israel, Nigeria and France owing to inactivation of the PhoP/PhoQ regulator mgrB: an epidemiological and molecular study. Int. J. Antimicrob. Agents. [Epub ahead of print]. 10.1016/j.ijantimicag.2014.07.020 25264127

[B150] Ortega X. P. Cardona S. T. Brown A. R. Loutet S. A. Flannagan R. S. Campopiano D. J.. (2007). A putative gene cluster for aminoarabinose biosynthesis is essential for Burkholderia cenocepacia viability. J. Bacteriol. 189, 3639–3644. 10.1128/JB.00153-07 17337576 PMC1855895

[B105] Owusu-Anim D. Kwon D. H. (2012). Differential role of two-component regulatory systems (phoPQ and pmrAB) in polymyxin B susceptibility of *Pseudomonas aeruginosa*. Adv. Microbiol. 2. 10.4236/aim.2012.21005 24349887 PMC3859615

[B106] Padilla E. Llobet E. Domenech-Sanchez A. Martinez-Martinez L. Bengoechea J. A. Alberti S. (2010). *Klebsiella pneumoniae* AcrAB efflux pump contributes to antimicrobial resistance and virulence. Antimicrob. Agents Chemother. 54, 177–183. 10.1128/AAC.00715-09 19858254 PMC2798511

[B107] Park Y. K. Choi J. Y. Shin D. Ko K. S. (2011). Correlation between overexpression and amino acid substitution of the PmrAB locus and colistin resistance in *Acinetobacter baumannii*. Int. J. Antimicrob. Agents 37, 525–530. 10.1016/j.ijantimicag.2011.02.008 21497062

[B108] Pelletier M. R. Casella L. G. Jones J. W. Adams M. D. Zurawski D. V. Hazlett K. R.. (2013). Unique structural modifications are present in the lipopolysaccharide from colistin-resistant strains of *Acinetobacter baumannii*. Antimicrob. Agents Chemother. 57, 4831–4840. 10.1128/AAC.00865-13 23877686 PMC3811424

[B148] Poirel L. Jayol A. Bontron S. Villegas M.-V. Ozdamar M. Tükoglu S.. (2014). The mgrB gene as a key target for acquired resistance to colistin in Klebsiella pneumoniae. J. Antimicrob. Chemother. [Epub ahead of print]. 10.1093/jac/dku323 25190723

[B109] Pournaras S. Poulou A. Dafopoulou K. Chabane Y. N. Kristo I. Makris D.. (2014). Growth retardation, reduced invasiveness, and impaired colistin-mediated cell death associated with colistin resistance development in *Acinetobacter baumannii*. Antimicrob. Agents Chemother. 58, 828–832. 10.1128/AAC.01439-13 24247145 PMC3910856

[B110] Quesada A. Porrero M. C. Téllez S. Palomo G. García M. Domínguez L. (2014). Polymorphism of genes encoding PmrAB in colistin-resistant strains of *Escherichia coli* and *Salmonella enterica* isolated from poultry and swine. J. Antimicrob. Chemother. [Epub ahead of print]. 10.1093/jac/dku320 25150146

[B111] Raetz C. R. Reynolds C. M. Trent M. S. Bishop R. E. (2007). Lipid A modification systems in gram-negative bacteria. Annu. Rev. Biochem. 76, 295–329. 10.1146/annurev.biochem.76.010307.145803 17362200 PMC2569861

[B112] Reynolds C. M. Kalb S. R. Cotter R. J. Raetz C. R. (2005). A phosphoethanolamine transferase specific for the outer 3-deoxy-D-manno-octulosonic acid residue of *Escherichia coli* lipopolysaccharide. Identification of the eptB gene and Ca^2+^ hypersensitivity of an eptB deletion mutant. J. Biol. Chem. 280, 21202–21211. 10.1074/jbc.M500964200 15795227

[B113] Reynolds C. M. Ribeiro A. A. McGrath S. C. Cotter R. J. Raetz C. R. Trent M. S. (2006). An outer membrane enzyme encoded by *Salmonella* typhimurium lpxR that removes the 3′-acyloxyacyl moiety of lipid A. J. Biol. Chem. 281, 21974–21987. 10.1074/jbc.M603527200 16704973 PMC2702521

[B114] Rolain J. M. Diene S. M. Kempf M. Gimenez G. Robert C. Raoult D. (2013). Real-time sequencing to decipher the molecular mechanism of resistance of a clinical pan-drug-resistant *Acinetobacter baumannii* isolate from Marseille, France. Antimicrob. Agents Chemother. 57, 592–596. 10.1128/AAC.01314-12 23070160 PMC3535948

[B115] Rolain J. M. Roch A. Castanier M. Papazian L. Raoult D. (2011). *Acinetobacter baumannii* resistant to colistin with impaired virulence: a case report from France. J. Infect. Dis. 204, 1146–1147. 10.1093/infdis/jir475 21881132

[B116] Roland K. L. Martin L. E. Esther C. R. Spitznagel J. K. (1993). Spontaneous pmrA mutants of *Salmonella* typhimurium LT2 define a new two-component regulatory system with a possible role in virulence. J. Bacteriol. 175, 4154–4164. 8391535 10.1128/jb.175.13.4154-4164.1993 PMC204845

[B117] Rozalski A. Sidorczyk Z. Kotelko K. (1997). Potential virulence factors of *Proteus bacilli*. Microbiol. Mol. Biol. Rev. 61, 65–89. 9106365 10.1128/mmbr.61.1.65-89.1997 PMC232601

[B118] Samonis G. Korbila I. P. Maraki S. Michailidou I. Vardakas K. Z. Kofteridis D.. (2014). Trends of isolation of intrinsically resistant to colistin *Enterobacteriaceae* and association with colistin use in a tertiary hospital. Eur. J. Clin. Microbiol Infect. Dis. 33, 1505–1510. 10.1007/s10096-014-2097-8 24798249

[B119] Sassera D. Comandatore F. Gaibani P. D'Auria G. Mariconti M. Landini M. P. (2013). Comparative genomics of closely related strains of Klebsiella pneumoniae reveals genes possibly involved in colistin resistance. Ann. Microbiol. 64, 887–890 10.1007/s13213-013-0727-5

[B120] Schurek K. N. Sampaio J. L. Kiffer C. R. Sinto S. Mendes C. M. Hancock R. E. (2009). Involvement of pmrAB and phoPQ in polymyxin B adaptation and inducible resistance in non-cystic fibrosis clinical isolates of *Pseudomonas aeruginosa*. Antimicrob. Agents Chemother. 53, 4345–4351. 10.1128/AAC.01267-08 19635950 PMC2764176

[B121] Sidorczyk Z. Zahringer U. Rietschel E. T. (1983). Chemical structure of the lipid A component of the lipopolysaccharide from a *Proteus mirabilis* Re-mutant. Eur. J. Biochem. 137, 15–22. 10.1111/j.1432-1033.1983.tb07789.x 6360683

[B122] Silipo A. Molinaro A. Cescutti P. Bedini E. Rizzo R. Parrilli M.. (2005). Complete structural characterization of the lipid A fraction of a clinical strain of B. cepacia genomovar I lipopolysaccharide. Glycobiology 15, 561–570. 10.1093/glycob/cwi029 15610978

[B123] Snitkin E. S. Zelazny A. M. Gupta J. Palmore T. N. Murray P. R. Segre J. A. (2013). Genomic insights into the fate of colistin resistance and *Acinetobacter baumannii* during patient treatment. Genome Res. 23, 1155–1162. 10.1101/gr.154328.112 23564252 PMC3698508

[B124] Snitkin E. S. Zelazny A. M. Thomas P. J. Stock F. Henderson D. K. Palmore T. N.. (2012). Tracking a hospital outbreak of carbapenem-resistant *Klebsiella pneumoniae* with whole-genome sequencing. Sci. Transl. Med. 4, 148ra116. 10.1126/scitranslmed.3004129 22914622 PMC3521604

[B125] Srinivasan V. B. Rajamohan G. (2013). KpnEF, a new member of the *Klebsiella pneumoniae* cell envelope stress response regulon, is an SMR-type efflux pump involved in broad-spectrum antimicrobial resistance. Antimicrob. Agents Chemother. 57, 4449–4462. 10.1128/AAC.02284-12 23836167 PMC3754300

[B126] Stein A. Raoult D. (2002). Colistin: an antimicrobial for the 21st century? Clin. Infect. Dis. 35, 901–902. 10.1086/342570 12228836

[B127] Sun S. Negrea A. Rhen M. Andersson D. I. (2009). Genetic analysis of colistin resistance in *Salmonella enterica* serovar Typhimurium. Antimicrob. Agents Chemother. 53, 2298–2305. 10.1128/AAC.01016-08 19332669 PMC2687247

[B128] Tamayo R. Choudhury B. Septer A. Merighi M. Carlson R. Gunn J. S. (2005a). Identification of cptA, a PmrA-regulated locus required for phosphoethanolamine modification of the *Salmonella enterica* serovar typhimurium lipopolysaccharide core. J. Bacteriol. 187, 3391–3399. 10.1128/JB.187.10.3391-3399.2005 15866924 PMC1112023

[B129] Tamayo R. Prouty A. M. Gunn J. S. (2005b). Identification and functional analysis of *Salmonella enterica* serovar Typhimurium PmrA-regulated genes. FEMS Immunol. Med. Microbiol. 43, 249–258. 10.1016/j.femsim.2004.08.007 15681155

[B130] Touze T. Tran A. X. Hankins J. V. Mengin-Lecreulx D. Trent M. S. (2008). Periplasmic phosphorylation of lipid A is linked to the synthesis of undecaprenyl phosphate. Mol. Microbiol. 67, 264–277. 10.1111/j.1365-2958.2007.06044.x 18047581 PMC2229476

[B131] Tran A. X. Lester M. E. Stead C. M. Raetz C. R. Maskell D. J. McGrath S. C.. (2005). Resistance to the antimicrobial peptide polymyxin requires myristoylation of *Escherichia coli* and *Salmonella* typhimurium lipid A. J. Biol. Chem. 280, 28186–28194. 10.1074/jbc.M505020200 15951433

[B132] Trent M. S. Pabich W. Raetz C. R. Miller S. I. (2001a). A PhoP/PhoQ-induced Lipase (PagL) that catalyzes 3-O-deacylation of lipid A precursors in membranes of *Salmonella* typhimurium. J. Biol. Chem. 276, 9083–9092. 10.1074/jbc.M010730200 11108722

[B133] Trent M. S. Ribeiro A. A. Lin S. Cotter R. J. Raetz C. R. (2001b). An inner membrane enzyme in *Salmonella* and *Escherichia coli* that transfers 4-amino-4-deoxy-L-arabinose to lipid A: induction on polymyxin-resistant mutants and role of a novel lipid-linked donor. J. Biol. Chem. 276, 43122–43131. 10.1074/jbc.M106961200 11535604

[B134] Vaara M. Vaara T. Jensen M. Helander I. Nurminen M. Rietschel E. T.. (1981). Characterization of the lipopolysaccharide from the polymyxin-resistant pmrA mutants of *Salmonella* typhimurium. FEBS Lett. 129, 145–149. 10.1016/0014-5793(81)80777-6 6268456

[B135] Velkov T. Deris Z. Z. Huang J. X. Azad M. A. K. Butler M. Sivanesan S.. (2013a). Surface changes and polymyxin interactions with a resistant strain of *Klebsiella pneumoniae*. Innate Immun. 20, 350–363. 10.1177/1753425913493337 23887184 PMC4242413

[B136] Velkov T. Soon R. L. Chong P. L. Huang J. X. Cooper M. A. Azad M. A.. (2013b). Molecular basis for the increased polymyxin susceptibility of *Klebsiella pneumoniae* strains with under-acylated lipid A. Innate Immun. 19, 265–277. 10.1177/1753425912459092 23008349 PMC4242410

[B137] Vinogradov E. Lindner B. Seltmann G. Radziejewska-Lebrecht J. Holst O. (2006). Lipopolysaccharides from *Serratia marcescens* possess one or two 4-amino-4-deoxy-L-arabinopyranose 1-phosphate residues in the lipid A and D-glycero-D-talo-oct-2-ulopyranosonic acid in the inner core region. Chemistry 12, 6692–6700. 10.1002/chem.200600186 16807947

[B138] Wang W. B. Chen I. C. Jiang S. S. Chen H. R. Hsu C. Y. Hsueh P. R.. (2008). Role of RppA in the regulation of polymyxin b susceptibility, swarming, and virulence factor expression in *Proteus mirabilis*. Infect. Immun. 76, 2051–2062. 10.1128/IAI.01557-07 18316383 PMC2346679

[B139] Weiss J. Victor M. Cross A. S. Elsbach P. (1982). Sensitivity of K1-encapsulated *Escherichia coli* to killing by the bactericidal/permeability-increasing protein of rabbit and human neutrophils. Infect. Immun. 38, 1149–1153. 6759406 10.1128/iai.38.3.1149-1153.1982 PMC347869

[B140] Winfield M. D. Groisman E. A. (2004). Phenotypic differences between *Salmonella* and *Escherichia coli* resulting from the disparate regulation of homologous genes. Proc. Natl. Acad. Sci. U.S.A. 101, 17162–17167. 10.1073/pnas.0406038101 15569938 PMC534605

[B141] Wosten M. M. Groisman E. A. (1999). Molecular characterization of the PmrA regulon. J. Biol. Chem. 274, 27185–27190. 10.1074/jbc.274.38.27185 10480935

[B142] Yan A. Guan Z. Raetz C. R. (2007). An undecaprenyl phosphate-aminoarabinose flippase required for polymyxin resistance in *Escherichia coli*. J. Biol. Chem. 282, 36077–36089. 10.1074/jbc.M706172200 17928292 PMC2613183

[B143] Yethon J. A. Gunn J. S. Ernst R. K. Miller S. I. Laroche L. Malo D.. (2000). *Salmonella enterica* serovar typhimurium waaP mutants show increased susceptibility to polymyxin and loss of virulence *In vivo*. Infect. Immun. 68, 4485–4491. 10.1128/IAI.68.8.4485-4491.2000 10899846 PMC98355

[B144] Young M. L. Bains M. Bell A. Hancock R. E. (1992). Role of *Pseudomonas aeruginosa* outer membrane protein OprH in polymyxin and gentamicin resistance: isolation of an OprH-deficient mutant by gene replacement techniques. Antimicrob. Agents Chemother. 36, 2566–2568. 10.1128/AAC.36.11.2566 1336952 PMC284378

[B145] Zhou Z. Ribeiro A. A. Lin S. Cotter R. J. Miller S. I. Raetz C. R. (2001). Lipid A modifications in polymyxin-resistant *Salmonella* typhimurium: PMRA-dependent 4-amino-4-deoxy-L-arabinose, and phosphoethanolamine incorporation. J. Biol. Chem. 276, 43111–43121. 10.1074/jbc.M106960200 11535603

